# Improving the Stability of Colloidal CsPbBr_3_ Nanocrystals with an Alkylphosphonium Bromide as Surface Ligand Pair

**DOI:** 10.1021/acsenergylett.5c00124

**Published:** 2025-04-11

**Authors:** Meenakshi Pegu, Hossein Roshan, Clara Otero-Martínez, Luca Goldoni, Juliette Zito, Nikolaos Livakas, Pascal Rusch, Francesco De Boni, Francesco Di Stasio, Ivan Infante, Luca De Trizio, Liberato Manna

**Affiliations:** ^†^Nanochemistry, ^‡^Photonic Nanomaterials, ^§^Materials Characterization, ^○^Chemistry Facility, Istituto Italiano di Tecnologia, Via Morego 30, 16163 Genova, Italy; ∥ Dipartimento di Chimica e Chimica Industriale, Universitá di Genova,16146 Genova, Italy; ⊥ BCMaterials, Basque Center for Materials, Applications, and Nanostructures, UPV/EHU Science Park, Leioa 48940, Spain; □ Ikerbasque Basque Foundation for Science, Bilbao 48009, Spain

## Abstract

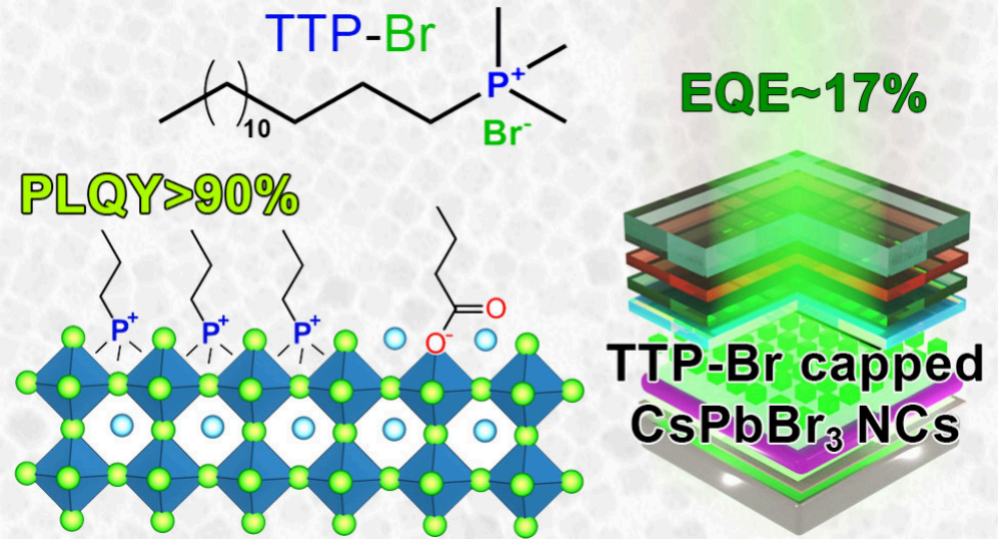

In this study, we synthesized a phosphonium-based ligand, trimethyl(tetradecyl)phosphonium bromide (TTP-Br), and employed it in the postsynthesis surface treatment of Cs-oleate-capped CsPbBr_3_ nanocrystals (NCs). The photoluminescence quantum yield (PLQY) of the NCs increased from ∼60% to more than 90% as a consequence of replacing Cs-oleate with TTP-Br ligand pairs. Density functional theory calculations revealed that TTP^+^ ions bind to the NC surface by occupying Cs^+^ surface sites and orienting one of their P–CH_3_ bonds perpendicular to the surface, akin to quaternary ammonium passivation. Importantly, TTP-Br-capped NCs exhibited higher stability in air compared to didodecyldimethylammonium bromide-capped CsPbBr_3_ NCs (which are considered a benchmark system), retaining ∼90% of their PLQY after 6 weeks of air exposure. Light-emitting diodes fabricated with TTP-Br-capped NCs achieved a maximum external quantum efficiency of 17.2%, demonstrating the potential of phosphonium-based molecules as surface ligands for CsPbBr_3_ NCs in optoelectronic applications.

Colloidal nanocrystals (NCs) of lead halide perovskites, with chemical formula CsPbX_3_ (X = Cl, Br, I), have emerged as promising active elements for optoelectronic devices, including light-emitting diodes (LEDs), displays, scintillators, solar concentrators, photodetectors, and solar cells.^1−3^ Such interest stems from the remarkable optical properties of these NCs, which include high photoluminescence (PL) quantum yield (QY), narrow PL line widths, and tunable optical band gaps that extend from the visible spectral region up to the near-infrared.^4−10^ These NCs, however, suffer from two main drawbacks due to their strong ionic character: (i) their inherent high solubility in polar solvents makes them prone to partial dissolution or etching when exposed to moisture, air, or during standard purification procedures, and (ii) the surface ligands are typically weakly bound and tend to desorb from the surface. The latter point is critical, since even a partial desorption of surface ligands not only negatively affects the colloidal stability of CsPbX_3_ NCs but also contributes to the degradation of their optical properties.^11−13^

In this context, typical surface ligands used in the synthesis of CsPbX_3_ NCs are alkylamines and/or carboxylic acids, which bind to the NCs’ surface as ion pairs, specifically as alkylammonium-halides and Cs-carboxylates,^14,15^ with alkylammonium cations occupying surface Cs^+^ sites and carboxylate anions replacing surface halide anions. These charged ligands can easily detach from the surface of CsPbX_3_ NCs upon protonation or deprotonation, for example by simply exposing the colloidal solution to air, eventually causing degradation in PLQY.^13,16^ To address these issues and improve both the colloidal stability and PLQY of CsPbX_3_ NCs, various alternative surface ligands have been explored so far. The choice usually falls on molecules such as alkyl sulfonates or phosphonates^17,18^ that have a higher binding strength to the surface of the NCs compared to alkylammonium-halide and Cs-carboxylate, or molecules that are unaffected by protonation or deprotonation, such as alkyl quaternary ammonium halides or zwitterionic molecules.^16,19−22^ We cite here only some representative examples: Cai et al. successfully employed several alkyl sulfonium bromides for the colloidal synthesis of CsPbBr_3_ NCs,^23^ while Imran et al. replaced Cs-oleate ion couples on the surface of CsPbBr_3_ NCs with didodecyldimethylammonium bromide (DDA-Br).^16^ Kreig et al. reported an effective approach to synthesize CsPbBr_3_ NCs using zwitterionic long-chain molecules such as sulfobetaine, phosphocholine, or γ-amino acid.^21^ Two common features of CsPbBr_3_ NCs from all these works are a near-unity PLQY and improved stability under ambient conditions and even upon washing with solvents to remove excess ligands.

Another unexplored class of ligands with the potential to deliver efficient and stable CsPbBr_3_ NCs and deserving further investigation are the alkylphosphonium salts. Large aromatic phosphonium halide ion pairs have been used to treat CsPbX_3_ NC films, enabling effective charge injection and mobility and thereby improving the efficiency of the corresponding perovskite-based LED (Table S1).^24−26^ Despite these advancements, no in-depth investigation has yet been conducted to determine whether these species can efficiently bind to the surface of CsPbBr_3_ NCs. Given the structural similarity between quaternary phosphonium halides and quaternary ammonium halides, we hypothesize that the quaternary phosphonium halides as well should also be capable of anchoring to the surface of CsPbBr_3_ NCs without altering the NCs’ morphology and potentially resulting in stable and strongly emissive NCs. To test this hypothesis, we synthesized trimethyl(tetradecyl)phosphonium bromide (TTP-Br), a quaternary alkyl phosphonium halide salt in which the four organic substituents bound to the phosphorus atom are three methyl groups and a long tetradecyl group, and evaluated its effectiveness as a surface ligand for CsPbBr_3_ NCs.^27^ This compound, which has the advantage of a relatively accessible phosphonium group while at the same time being soluble in most common organic solvents, was employed in a postsynthesis ligand exchange procedure involving Cs-oleate-capped CsPbBr_3_ NCs in toluene (Scheme 1).

**Scheme 1:**
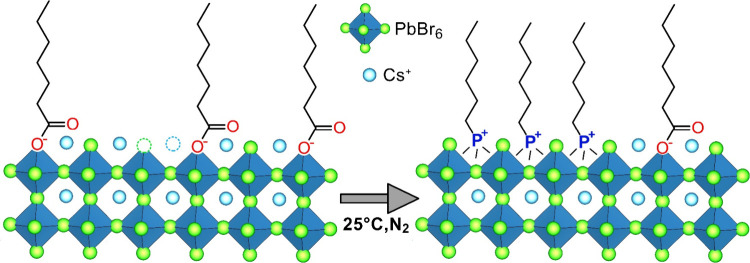
Sketch of the Post-Synthesis Ligand Exchange Process Involving Treatment of Cs-Oleate-Capped CsPbBr_3_ NCs with TTP-Br

Our results demonstrated a successful exchange of Cs-oleate for TTP-Br, with the PLQY of CsPbBr_3_ NCs increasing from 62% (as-synthesized NCs, coated with Cs-oleate) to over 90% (TTP-Br coated NCs). Nuclear magnetic resonance (NMR) analyses revealed that the ligand shell of the final NCs comprised 92% TTP-Br (with a density of 1.28 ligands/nm^2^) and a residual 8% of Cs-oleate. To rationalize these findings, we performed density functional theory (DFT) calculations, which revealed that TTP-Br has a high binding energy to the surface of CsPbBr_3_ NCs (42.73 kcal/mol), a value comparable to that of DDA-Br.^11^

To evaluate whether this quaternary phosphonium salt could provide better surface passivation for CsPbBr_3_ NCs compared to quaternary alkylammonium-based salts, we also performed the same ligand exchange procedure using the corresponding ammonium bromide pair, that is, trimethyl(tetradecyl)ammonium bromide (TTN-Br). Surprisingly, ligand exchange with TTN-Br led to the precipitation of CsPbBr_3_ NCs, which could not be redispersed in any organic solvent. Therefore, we decided to compare the stability of TTP-Br-capped CsPbBr_3_ NCs to that of DDA-Br-capped NCs, which are widely regarded as a benchmark system due to their near unity PLQY and high colloidal stability under various ambient stimuli such as heat, solvent washing, and upon long-term storage.^16,28^ Additionally, the ligand exchange procedure that we employed to prepare TTP-Br-capped NCs is analogous to the one reported to prepare DDA-Br-capped NCs, with the starting point being Cs-oleate-capped CsPbBr_3_ NCs in both cases.^16^ This allows for a clear comparison between the two ligands, as the two end products feature the same inorganic core but different ligand shells.

The comparison was done by exposing both NC solutions to air for a time span of 6 weeks, during which the TTP-Br-capped NCs exhibited higher stability compared to DDA-Br-capped NCs, retaining ∼ 90% of their initial PLQY at the end of this time span, while the DDA-Br-capped NCs retained only ∼ 76% of their initial PLQY. The higher air stability of the TTP-Br-capped NCs, along with the fact that TTP-Br ligands are less bulky and, therefore, likely more electrically conductive than DDA-Br (DDA^+^ has two dodecyl alkyl chains, while TTP^+^ has only one tetradecyl alkyl chain), motivated us to fabricate green-emitting LED devices with both TTP-Br-capped and DDA-Br-capped NCs and make a comprehensive comparison. The LEDs with TTP-Br-capped NCs achieved a maximum external quantum efficiency (EQE) of 17.2% at high luminance of 2600 cd m^–2^, significantly surpassing the EQE of LED with DDA-Br-capped NCs, which had 7.4% EQE. Moreover, stability tests under operation conditions showed higher operational stability of TTP-Br LEDs in comparison with the DDA-Br-capped counterparts.

We synthesized TTP-Br using a one-pot synthesis approach, which involves the nucleophilic substitution reaction of trimethylphosphine with tetradecyl bromide (eq 1, Scheme S1) with a reaction yield of ∼ 75% (see the Experimental Section, Figures S1–S3 and Scheme S1 of the Supporting Information, SI, for details).
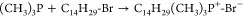
1

The purified TTP-Br salt is soluble at room temperature in various organic solvents, including chloroform and partially in toluene, therefore it could be readily employed in a ligand-exchange procedure involving Cs-oleate-capped CsPbBr_3_ NCs. Cs-oleate-capped CsPbBr_3_ NCs were synthesized following the well-established hot injection protocol reported by Imran et al. with minor modifications (see the Experimental Section).^16,29^ The ligand exchange process was performed as follows: after quenching the synthesis of Cs-oleate-capped NCs, 2 mL of a 25 mM solution of TTP-Br in chloroform/toluene (10% v/v) was added to a portion of the crude reaction solution (3 mL) containing 0.09 mmol of CsPbBr_3_ NCs. The reaction was allowed to proceed for 20 min under a nitrogen atmosphere, after which the product was washed with ethyl acetate and redispersed in toluene (see the Experimental Section for more details).

Upon ligand exchange with TTP-Br, the NCs retained their cubic shape, with a slight size reduction from 9.7 ± 1.2 nm (Figure S4a) to 8.9 ± 1.6 nm (Figure 1a), indicating a minor etching of the NCs, compatible with what was reported for the DDA-Br case.^16^ X-ray diffraction (XRD) patterns of the CsPbBr_3_ NCs before and after the exchange featured the characteristic peaks of the CsPbBr_3_ orthorhombic phase (ICSD Number 243735) at 15.05°, 21.54°, and 30.56°, corresponding to the (100), (110), and (200) lattice planes, respectively (Figures 1b and S4b). To further investigate the effects of the ligand exchange procedure, we performed X-ray photoelectron spectroscopy (XPS) measurements. Upon exchange with TTP-Br, the relative atomic percentage of O decreased from 9.3% to 2.8%, while the Br/Pb atomic ratio increased from 2.28 to 2.75 (Table S2 and Figures S5 and S6). Moreover, the final sample exhibited additional XPS peaks at 132.6 and 133.4 eV, ascribed to P *2p*, corroborating the effective anchoring of TTP-Br onto the NCs’ surface (Figure 1c). The presence of homogeneously distributed P on the TTP-Br-exchanged CsPbBr_3_ NCs was further confirmed by transmission electron microscopy (TEM) and scanning electron microscopy (SEM) energy-dispersive X-ray spectroscopy (EDX) mapping (Figures S7 and S8 and Tables S3 and S4).

**Figure 1:**
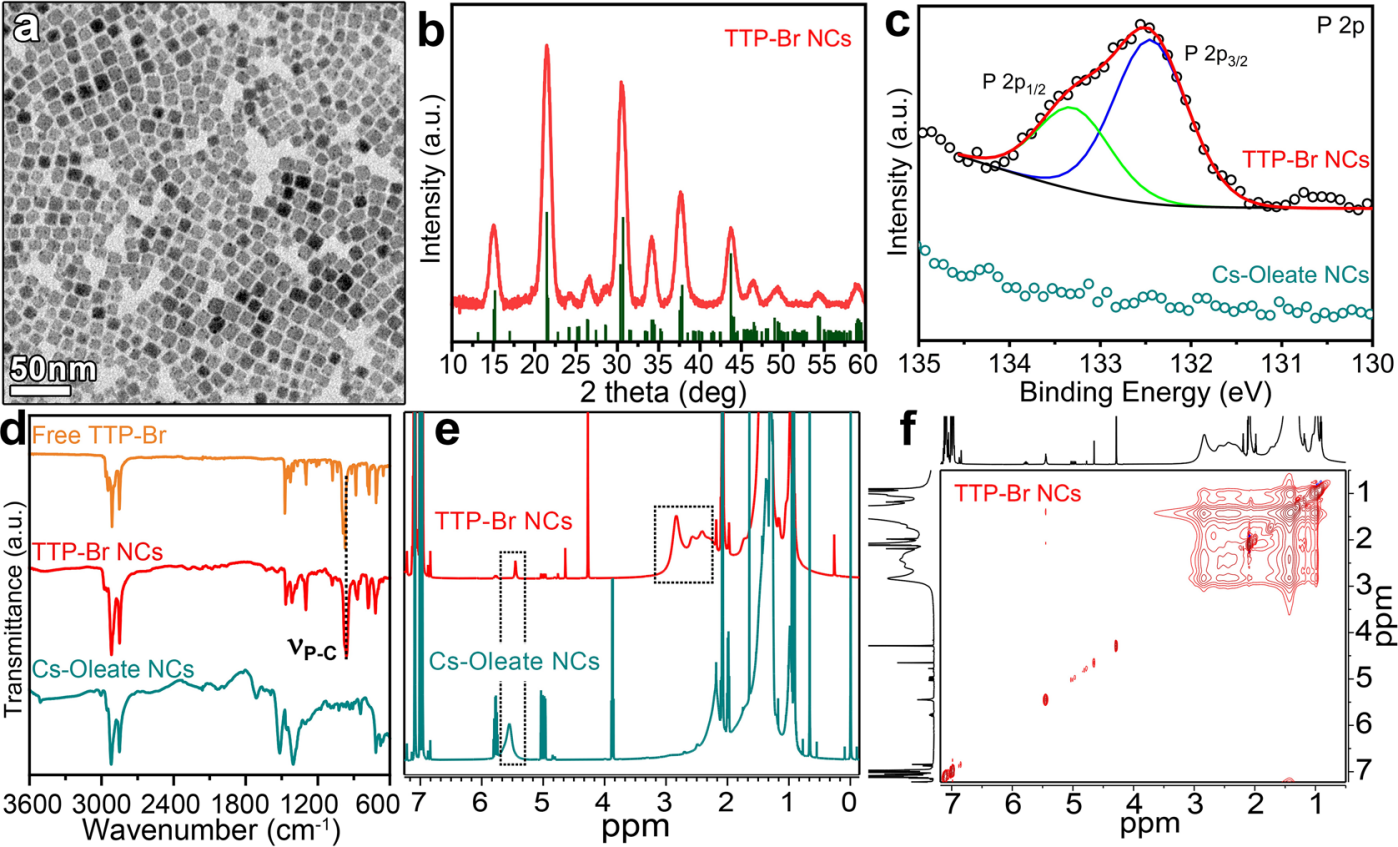
(a) TEM micrograph of TTP-Br-capped CsPbBr_3_ NCs. (b) X-ray diffraction pattern of TTP-Br-capped CsPbBr_3_ NCs and reference CsPbBr_3_ orthorhombic bulk reflections (ICSD Number 243735). (c) XPS P*2p* spectra of TTP-Br- and Cs-Oleate-capped CsPbBr_3_ NCs. (d) FTIR spectra of the TTP-Br ligand and TTP-Br- and Cs-oleate-capped CsPbBr_3_ NCs. (e) ^1^H NMR spectra at 298 K of Cs-oleate- and TTP-Br-capped CsPbBr_3_ NCs (see the Supporting Information for more details). (f) 2D NOESY spectrum of TTP-Br-capped CsPbBr_3_ NCs at 313 K (see Supporting Information for the detailed spectrum).

To assess the degree of the Cs-oleate → TTP-Br exchange and to reveal the ligand shell composition of the exchanged NCs, we performed both Fourier-transform Infrared (FTIR) and NMR analyses. The FTIR spectra of the NCs before and after the TTP-Br exchange revealed a significant replacement of oleate species with TTP-Br: (i) a reduction in the peaks at 1710 and 1535 cm^–1^ corresponding to the asymmetric and symmetric stretching vibrations of the C=O and COO^–^ groups, respectively (Figure S9); (ii) the emergence of a peak at approximately 990 cm^–1^, attributable to the P–C stretching vibrations (Figure 1d).^27,30^ Similarly, the ^1^H NMR spectra of CsPbBr_3_ NCs before and after ligand exchange with TTP-Br showed a reduction of the signal at 5.48 ppm, ascribed to the protons of the double bond in the oleate species, along with the appearance of broad peaks between 2.01 and 2.84 ppm (Figure 1e). Such peaks were attributed to the Me_3_-P groups of TTP-Br bound to the surface of the NCs (see Figures S1–S3 and Figures S10–S12 for complete signal assignments of the TTP-Br ligand and TTP-Br-capped CsPbBr_3_ NCs). At 298 K, the free TTP-Br ligand was observed to form micelles or aggregates in toluene-*d*, as indicated by the ^1^H–^1^H NOESY spectrum (Figure S13). To ensure complete solubility of TTP-Br and accurate assignment of the peaks on the NCs’ surface, NMR measurements of the TTP-Br ligand as well as TTP-Br-capped NCs were conducted at 313 K (Figure S14–S16). The dynamic interaction of the TTP-Br ligand with the NCs’ surface was demonstrated by ^1^H–^1^H NOESY at 313 K,^19^ which evidenced negative (red) NOE cross peaks for the TTP-Br-capped NCs (Figure 1f), typical of species with a long correlation time (τ_c_), i.e. with a slow tumbling regime in solution. Positive (blue) NOE cross-peaks were observed for the free TTP-Br at 313 K, characteristic of a molecule with the fast-tumbling regime in solution (Figure S15). To quantify the extent of Cs-oleate → TTP-Br replacement, we performed a quantitative NMR (*q*-NMR) analysis of the final NC sample dissolved in deuterated dimethyl sulfoxide (Figure S17), coupled with elemental analysis of the same solution performed via inductively coupled plasma optical emission spectroscopy (ICP-OES).^31,32^ Our results indicate that 92% of the ligand shell was composed of TTP-Br, while the remaining 8% consisted of Cs-oleate, with a calculated surface density of 1.28 TTP-Br molecules per nm^2^ and 0.10 oleate species per nm^2^ (see Table S5 in the Supporting Information for details).

In order to assess the potential advantages of using TTP-Br compared to quaternary alkyl ammonium salts in the passivation of CsPbBr_3_ NCs, we carried out the same ligand exchange procedure using trimethyl(tetradecyl)ammonium bromide (TTN-Br), the ammonium analogue of TTP-Br. Unfortunately, the NCs precipitated during the exchange and the collected product could not be redispersed in toluene nor in chloroform. Therefore, we decided to compare the TTP-Br-capped NCs with DDA-Br-capped ones, the latter representing one of the best CsPbBr_3_ NC systems regarding PLQY and colloidal/air stability.^11^ DDA-Br-capped NCs were prepared using the same ligand exchange protocol employed for TTP-Br-capped NCs, starting from Cs-oleate-capped NCs (see the Experimental Section for details). The resulting DDA-Br-capped NCs had a size of 8.8 ± 1.9 nm, with XRD patterns corresponding to the orthorhombic phase of the CsPbBr_3_ NCs (Figure S18). Similar to our previous reports, XPS and FTIR analyses confirmed the surface binding of the DDA-Br ligand on the NCs (Table S2, Figures S19 and S20).^16^ The NMR measurements confirmed the effective binding of DDA-Br ligand on the NCs’ surface (Figures S21–S25), with DDA-Br and Cs-oleate coverage of 1.32 and 0.27 ligands/nm^2^, respectively (Table S5 and Figure S26).^16,29^

In terms of optical properties, both TTP-Br- and DDA-Br-capped NCs exhibited a slightly blue-shifted PL and excitonic absorption peaks with respect to the initial Cs-oleate-capped NCs (Table 1, Figure 2a), consistent with the slight reduction in size observed after the exchange in both cases. The PLQY increased from 62 ± 6% to 91 ± 9% in the case of TTP-Br-capped NCs and to 98 ± 9% in the case of DDA-Br-capped NCs (Table 1). PL decay measurements revealed that the PL lifetime (**τ**_**avg**_) increased in solution for both the DDA-Br and TTP-Br samples, from 8.86 ns (for Cs-oleate-capped NCs) to 10.74 and 11.29 ns, respectively (Figure 2b, Figure S27, and Table S6). As these increases in PL lifetime go hand in hand with increases in PLQY, we conclude that fast, nonradiative recombination is significantly reduced in these ligands exchanged NCs, most likely due to more effective surface passivation. PL measurements performed in thin films at temperatures ranging from 7 K to 267 K indicated that both DDA-Br and TTP-Br-capped NCs featured the typical temperature-dependent behavior of lead halide perovskite NCs, that is, (i) a blue shift of the PL emission and (ii) an increase in PL lifetime when raising the temperature from 7 K to 267 K (see Figure S28 and Tables S7 and S8 of the Supporting Information for details).^33,34^ The temperature-dependent blue-shift is attributed to the thermal expansion of the perovskite lattice,^33^ which reduces the overlap between the Pb 6s and Br 4p orbitals and results in a lower energy of the antibonding crystal orbital at the valence band maximum, leading to an increase in the bandgap energy.^34,35^ The shorter average lifetime at lower temperatures is consistent with the exciton ground state being a bright triplet for NCs of sizes around 9–10 nm (as those studied in this work) or beyond, in agreement with previous experimental and theoretical works.^10,36,37^

**Figure 2:**
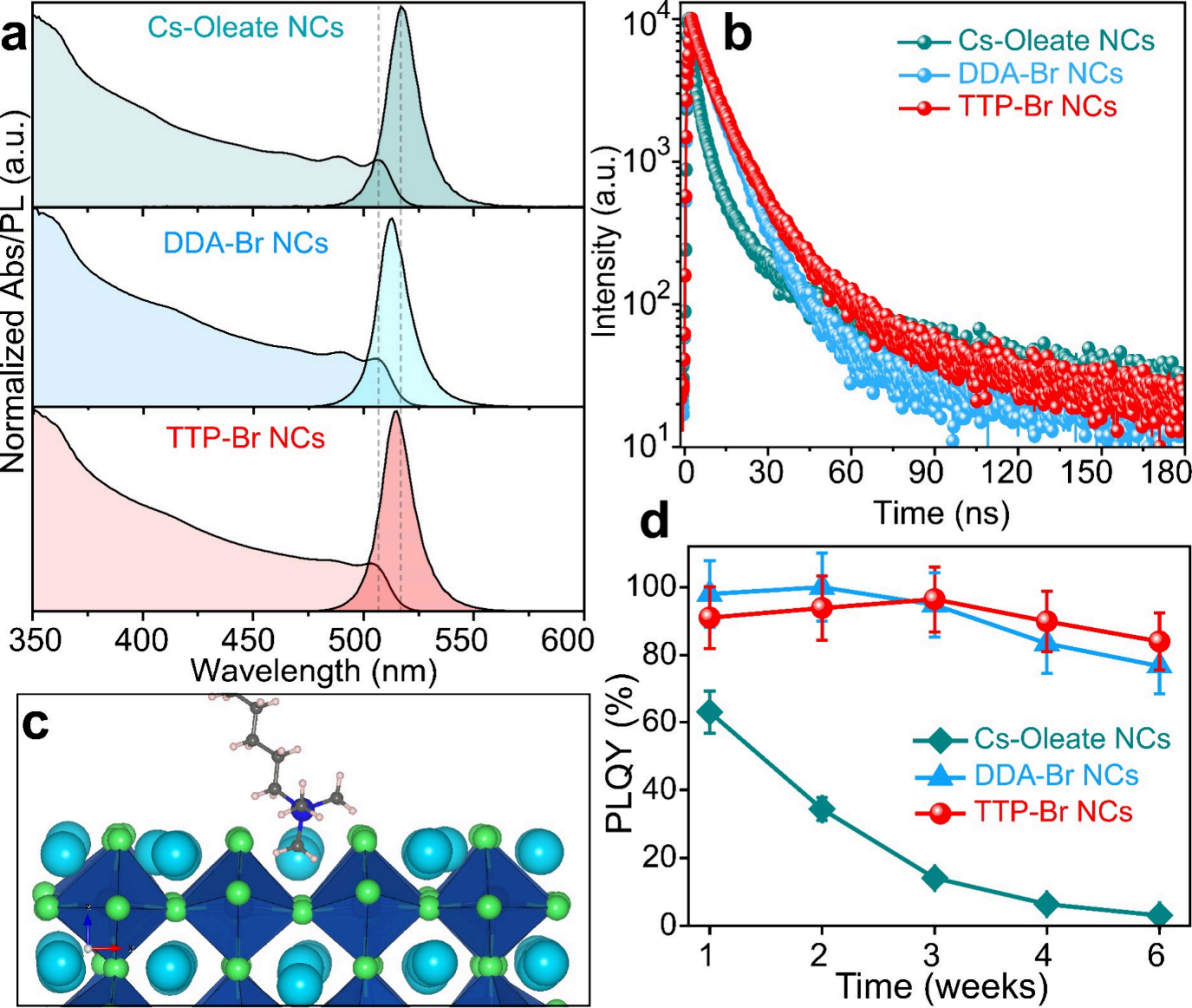
(a) UV–visible absorption and PL spectra of Cs-oleate-, DDA-Br-, and TTP-Br-capped CsPbBr_3_ NCs. (b) PL decay profile of Cs-oleate-, DDA-Br-, and TTP-Br-capped CsPbBr_3_ NCs. (c) Binding configuration of TTP-Br- ligands sitting in the A-site of the CsPbBr_3_ NCs’ surface. (d) PLQY stability of Cs-oleate-, DDA-Br-, and TTP-Br-capped CsPbBr_3_ NCs over time at ambient storage conditions.

**Table 1:** Optical Properties of the Different NC Samples in Solution at Room Temperature

NCs sample	Abs_max_ (nm)	PL_max_ (nm)	fwhm (nm)	PLQY (%) week 1	PLQY (%) week 2	τ_avg_ (ns)
Cs-oleate	508	517	18.5	62 ± 6	36 ± 4	8.86
DDA-Br	505	512	17.2	∼98	∼100	10.74
TTP-Br	506	514	18.0	91 ± 9	96 ± 9	11.29

To evaluate the stability of all the samples, we exposed NC dispersions in toluene to air for a period of 6 weeks. Among the three samples, TTP-Br-capped NCs exhibited the best stability featuring a PLQY of ∼85% at the end of the test, while DDA-Br- and Cs-oleate-capped NCs exhibited a PLQY of ∼76 and ∼3%, respectively (Figure 2d). Both the TTP-Br- and DDA-Br-capped NCs were observed to slightly increase in size from 9.8 ± 1.8 nm to 10.3 ± 1.6 nm after 6 weeks of exposure to air (Figure S29), likely due to a slow Oswald ripening process. XRD patterns of DDA-Br and TTP-Br-capped NCs exposed to air after 6 weeks were compatible with the presence of the orthorhombic phase of CsPbBr_3_ crystal structure, with no evidence of secondary phases, indicating good structural stability in both samples (Figure S30). Quaternary phosphonium/ammonium ions have a stable positive charge, unlike, for example, primary ammonium or carboxylate ions, which can be instead deprotonated/protonated upon exposure to air (operated by humidity/water present in the air). This prevents these ligands from losing their charge, allowing them to continue binding to the surface of the NCs even upon exposure to air. We believe that this property enables these ligands to sufficiently protect the NCs from water/humidity, which is known to trigger the phase change from CsPbBr_3_ to Cs_4_PbBr_6._^12,16,38^ Indeed, even after extending their exposure to air for up to a period of two months and a half, both the TTP-Br-capped and DDA-Br-capped CsPbBr_3_ NCs did not transform, not even partially, into Cs_4_PbBr_6_, or into other phases, as proven by XRD analyses (Figure S31).

To better understand how phosphonium ligands bind to the surface of CsPbBr_3_ NCs and whether this differs significantly from quaternary ammonium ligands, we performed DFT calculations (details in the Experimental Section). We first examined the binding characteristics of a single ion pair on the NC surface. To do this, we constructed a cubic, charge-balanced CsPbBr_3_ NC model with a ∼ 2.4 nm edge-size and replaced one CsBr unit from the outer AX shell with either a TTP-Br or DDA-Br ion pair. Upon structural relaxation, we observed that the phosphonium group oriented one of its P–CH_3_ bonds perpendicular to the NC surface, similar to the orientation observed for the quaternary ammonium group (Figures 2c and S32).^16^ The calculated binding energies for TTP-Br (42.7 kcal/mol) and DDA-Br (45.2 kcal/mol) were also comparable and in line with reported values for primary alkylammonium bromide (45.3 kcal/mol), secondary alkylammonium bromide (48.2 kcal/mol), and zwitterionic ligands such as sulfobetaine (41.0 kcal/mol), at similar theoretical levels of calculations.^21,39^ This consistency has been ascribed to the dominance of electrostatic interactions in determining the binding strength with the perovskite NC surface, regardless of the type of anchoring group used. To investigate the enhanced PL and stability of NCs passivated by TTP-Br ligands, we replaced CsBr units on all six facets with alkyl phosphonium bromide pairs, achieving a surface concentration of 1.27 ligands/nm^2^—consistent with the experimental 1.28 ligands/nm^2^. To reduce system size and computational cost, we simplified TTP-Br by replacing the tetradecyl group with an ethyl group. As shown in Figure S33, the electronic structure remains free of midgap states, with a fully delocalized valence band maximum, similar to quaternary ammonium passivation.^16^ In contrast, purely Cs-oleate-capped NCs, as demonstrated by earlier DFT calculations,^19^ exhibit trap states at both high and low ligand concentrations, localized on the oxygen atoms in the carboxylate anchoring groups. This enables nonradiative recombination and contributes to the suboptimal PLQY observed in experiments. Ligand exchange with phosphonium or quaternary ammonium pairs effectively eliminates oxygen-related defects.

The improved air stability of TTP-Br-capped NCs, along with the fact that TTP-Br is less bulky and likely less electrically resistive than DDA-Br, could lead to improved charge injection. This motivated us to test these NCs in optoelectronic devices, specifically LEDs. The LEDs were prepared using a conventional structure in which the emissive layer is sandwiched between a hole transport layer (HTL) and an electron transport layer (ETL), with the HTL being deposited first. As HTL layers, we tested poly(triaryl amine) (PTAA) and polyvinyl carbazole (PVK), while as the ETL, we employed 2′,2′-(1,3,5-benzinetriyl)-tris(1-phenyl-1*H*-benzimidazole) (TPBi). We compared our devices with a double HTL (PTAA/PVK) architecture, namely, ITO/PEDOT:PSS/PTAA/PVK/CsPbBr_3_ NCs/TPBi/LiF/aluminum (Figure 3a), to those with a single HTL using either PTAA or PVK. Here, PEDOT is poly(3,4-ethylenedioxythiophene) and PSS is polystyrenesulfonate. A statistical EQE study demonstrated the significant superiority of double HTL LEDs over those with single HTL (Figure S34), showing that the double HTL architecture can achieve optimal band alignment and efficient hole injection into the emissive layer, thereby improving the charge balance in the active layer.^40^Figure 3b illustrates the band alignment of the emitting layer, as determined by UPS analysis and optical absorption spectra (Table S9 and Figures S35 and S36). It also depicts the energy levels of all other layers of the double HTL architecture, as well as those of the electrodes. The energy level values of ITO, PEDOT:PSS, PTAA, PVK, TPBi, and LiF/Aluminum layers (Figure 3b) were taken from our previous work.^41^ The electroluminescence (EL) spectrum of the LED showed an emission peak at 516 nm with a full width at half-maximum (fwhm) of 18 nm (∼84 meV), consistent across all voltages (Figure 3c). This matched the PL spectrum of the solid TTP-Br-capped NC film (PL peak at 514 with fwhm of ∼ 17.7 nm; thin film images of TTP-Br-capped and DDA-Br-capped NCs are presented in Figures S37 and S38), with only a minor red shift observed in the peak position of EL spectra (Figure 3c). No other EL peaks were observed, indicating the absence of deep traps or interlayer emission. The 1931 CIE diagram of the EL spectrum, obtained at a 6 V operating voltage (Figure S39), showed that the LED had CIE coordinates (*x* = 0.084, *y* = 0.775), indicating a high degree of saturation in the green region, which translated to a vibrant and intense green emission (see also the inset of Figure 3c). The gamut coverage of the green LED fell well within the Rec.709 color space, making it interesting for integration into displays. The LEDs statistically exhibit a turn-on voltage between 2.6 and 3 V, which is slightly higher than the bandgap energy of the emissive layer (CsPbBr_3_ NCs). The champion device demonstrated a luminance exceeding 10000 cd/m^2^ (Figure 3d) at a current density of 43 mA/cm^2^. The maximum EQE of the champion TTP-Br-capped NCs LED was 17.2%, and it was achieved at a luminance of 2600 cd/m^2^, corresponding to an operation voltage of 4.3 V and a current density of 5.8 mA/cm^2^ (Figure 3e). This relatively high EQE can be attributed to the high luminance of LEDs based on TTP-Br-capped NCs, which allowed efficient light emission while maintaining a relatively low current density.

**Figure 3:**
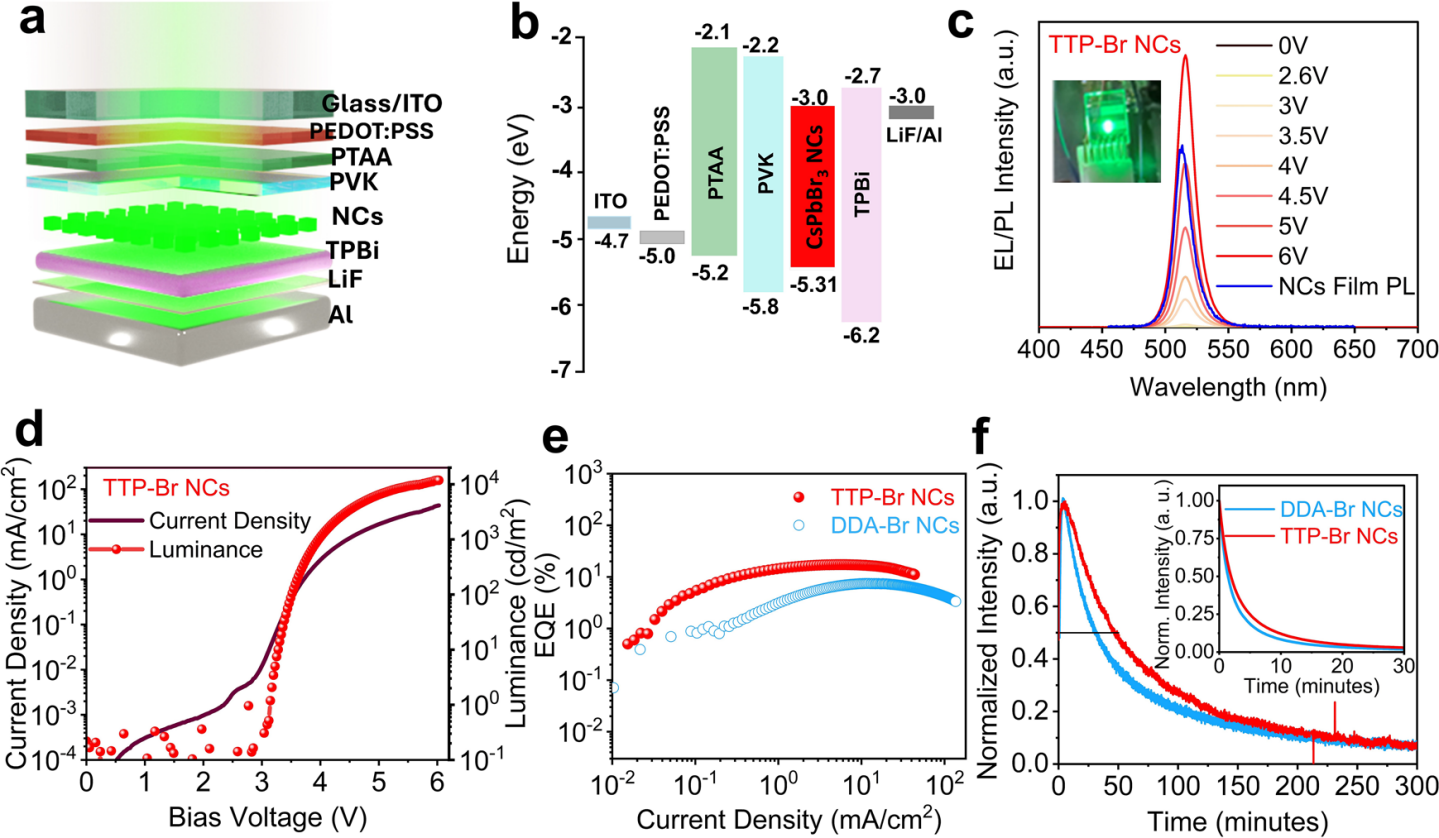
(a) Double HTL LED device configuration based on TTP-Br-capped CsPbBr_3_ NCs. (b) Band-energy alignment of TTP-Br-capped CsPbBr_3_ NCs with charge injection layers. (c) PL spectrum of TTP-Br-capped CsPbBr_3_ NC film and EL spectra of the double HTL LED at different applied voltages. (d) Current density and luminance versus driving voltage curves of the double HTL TTP-Br-capped CsPbBr_3_ NC-based champion LED device. (e) EQE versus current density of the double HTL LEDs champion devices based on TTP-Br-capped and DDA-Br-capped CsPbBr_3_ NCs. (f) Stability of the EL intensity under operation conditions with the initial luminance of 500 cd/m^2^, while the inset shows the intensity of light plotted over time with an applied bias of 7 V.

The performance of the TTP-Br-capped NC LEDs was compared to that of the DDA-Br-capped NC LEDs based on the very same architecture. The current density and luminance versus driving voltage curves for the double HTL-based DDA-Br-capped NC LED are presented in Figure S40. The maximum luminance achieved was 1313 cd/m^2^, while the EQE reached a maximum value of 7.4% (Figure 3e), consistent with what was reported in a previous work from our group on similar LEDs.^41^ It is worth highlighting that although our reported EQE is lower than the state-of-the-art value of 28.9%,^42^ it represents a significant improvement over the highest EQE values reported for LED based on NCs capped by long-chain ligands (∼15%, 9.8%, and 13.4%), specifically for DDA-Br-capped NCs.^28,41,43,44^ To investigate the origins of performance differences between TTP-Br-capped and DDA-Br-capped NC LEDs, we performed space-charge limited current (SCLC) measurements. The hole carrier mobility (μ) was determined for both DDA-Br-capped and TTP-Br-capped NCs-based devices by fitting their dark J-V curves to the SCLC model (Figure S41a,b, Table S10).^41,42^ The TTP-Br-capped NC-based device exhibited a higher hole mobility (1.43 × 10^–6^ cm^2^ V^–1^ s^–1^) compared to the DDA-Br-capped NCs (6.87 × 10^–7^ cm^2^ V^–1^ s^–1^). Moreover, the trap-filled limit voltage (*V*_TFL_) of the device based on TTP-Br-capped NCs (0.27 V) was lower than that of the device based on DDA-Br-capped NCs (0.36 V), suggesting that TTP-Br-capped NCs feature a lower density of surface traps and better surface passivation. Since both TTP-Br-capped and DDA-Br-capped samples exhibit comparable PLQY values, the superior charge transport in TTP-Br NCs likely contributes to the enhanced EQE observed in TTP-Br-based LEDs. The performance of the LEDs and the stability of the DDA-Br-capped and TTP-Br-capped NCs in solution motivated us to evaluate the luminance stability of the LEDs over time. To mitigate environmental effects, DDA-Br-capped and TTP-Br-capped NC LEDs were encapsulated with epoxy resin and tested under a constant applied voltage, corresponding to an initial luminance of 500 cd/m^2^ (Figure 3f). After an initial rise, the luminance began to degrade, and the performance was assessed using the half-lifetime (*T*_50_) criterion.^45^ The initial increase in light intensity during stability tests may result from ion migration, defect passivation, and charge redistribution, leading to a transient electroluminescence enhancement.^46^ The TTP-Br-capped NCs LED exhibited a *T*_50_ of 49 min, while the DDA-Br-capped NCs LED reached *T*_50_ after 30 min. Furthermore, we examined the LEDs stability under high-luminance conditions by applying a voltage of 7 V and monitoring the luminance decay over time (inset of Figure 3f). No significant differences between DDA-Br-capped and TTP-Br-capped NC LEDs were observed under high luminance, with both showing a *T*_50_ of only a few minutes.

In summary, we prepared trimethyl(tetradecyl)phosphonium bromide (TTP-Br), a quaternary alkyl phosphonium halide salt, and tested it in a postsynthesis ligand exchange procedure involving colloidal Cs-oleate-capped CsPbBr_3_ NCs. As a result of the Cs-oleate → TTP-Br exchange, the PLQY of CsPbBr_3_ NCs increased from 62% to 91%. NMR analyses confirmed the successful ligand replacement, indicating a final ligand shell composed of 92% TTP-Br (with a density of 1.28 ligands/nm^2^) and 8% Cs-oleate. The air stability of TTP-Br-capped NCs was compared to that of DDA-Br-capped NCs, which are considered a standard due to their high PLQY and air stability. After 6 weeks of air exposure, TTP-Br-capped NCs retained 90% of their PLQY, while the DDA-Br-capped NCs retained only 76%. DFT calculations revealed that TTP^+^ ions bind to the NCs’ surface by occupying A-sites and orienting one of their P–CH_3_ bonds perpendicular to the NC surface. The calculated binding energy is 42.7 kcal/mol, comparable to that of DDA-Br or alkylammonium-Br species in general. The higher air stability of TTP-Br-capped NCs compared to that of DDA-Br-capped NCs, along with the fact that TTP-Br ligands are less bulky and, therefore, likely less electrically resistive than DDA-Br, motivated us to fabricate green-emitting LED devices based on TTP-Br-capped NCs. Our LEDs achieved a maximum EQE of 17.2% at a luminance of 2600 cd m^–2^, surpassing the values reported to date for DDA-Br-based LED devices (∼15%). Our work demonstrates that alkylphosphonium salts represent a promising class of ligands for the surface passivation of perovskite NCs. Further engineering of the groups bound to the phosphonium head could potentially lead to optimized ligands capable of boosting the performance of perovskite-based optoelectronic devices.

## Supporting Information Available

Additional data, including synthesis scheme, TEM and STEM characterization, XRD analysis, XPS, SEM elemental mapping (EDS), elemental analysis, NMR and FTIR characterizations, optical characterizations, and UPS and DFT analyses are presented (PDF)

## References

[ref1] Shamsi J.; Urban A. S.; Imran M.; De Trizio L.; Manna L. Metal halide perovskite nanocrystals: synthesis, post-synthesis modifications, and their optical properties. Chem. Rev. 2019, 119 (5), 3296–3348. 10.1021/acs.chemrev.8b00644. 30758194 PMC6418875

[ref2] Bodnarchuk M. I.; Boehme S. C.; Ten Brinck S.; Bernasconi C.; Shynkarenko Y.; Krieg F.; Widmer R.; Aeschlimann B.; Günther D.; Kovalenko M. V.; Infante I. Rationalizing and controlling the surface structure and electronic passivation of cesium lead halide nanocrystals. ACS Energy Letters 2019, 4 (1), 63–74. 10.1021/acsenergylett.8b01669. 30662955 PMC6333230

[ref3] Byranvand M. M.; Otero-Martínez C.; Ye J.; Zuo W.; Manna L.; Saliba M.; Hoye R. L.; Polavarapu L. Recent progress in mixed a-site cation halide perovskite thin-films and nanocrystals for solar cells and light-emitting diodes. Advanced Optical Materials 2022, 10 (14), 2200423 10.1002/adom.202200423.

[ref4] Protesescu L.; Yakunin S.; Bodnarchuk M. I.; Krieg F.; Caputo R.; Hendon C. H.; Yang R. X.; Walsh A.; Kovalenko M. V. Nanocrystals of cesium lead halide perovskites (CsPbX_3_, X= Cl, Br, and I): novel optoelectronic materials showing bright emission with wide color gamut. Nano Lett. 2015, 15 (6), 3692–3696. 10.1021/nl5048779. 25633588 PMC4462997

[ref5] Liu D.; Guo Y.; Que M.; Yin X.; Liu J.; Xie H.; Zhang C.; Que W. Metal halide perovskite nanocrystals: application in high-performance photodetectors. Materials Advances 2021, 2 (3), 856–879. 10.1039/D0MA00796J.

[ref6] Yang D.; Cao M.; Zhong Q.; Li P.; Zhang X.; Zhang Q. All-inorganic cesium lead halide perovskite nanocrystals: synthesis, surface engineering and applications. Journal of Materials Chemistry C 2019, 7 (4), 757–789. 10.1039/C8TC04381G.

[ref7] Livakas N.; Toso S.; Ivanov Y. P.; Das T.; Chakraborty S.; Divitini G.; Manna L. CsPbCl_3_→CsPbI_3_ Exchange in Perovskite Nanocrystals Proceeds through a Jump-the-Gap Reaction Mechanism. J. Am. Chem. Soc. 2023, 145 (37), 20442–20450. 10.1021/jacs.3c06214. 37691231 PMC10515632

[ref8] Carey G. H.; Abdelhady A. L.; Ning Z.; Thon S. M.; Bakr O. M.; Sargent E. H. Colloidal quantum dot solar cells. Chem. Rev. 2015, 115 (23), 12732–12763. 10.1021/acs.chemrev.5b00063. 26106908

[ref9] Pan J.; Quan L. N.; Zhao Y.; Peng W.; Murali B.; Sarmah S. P.; Yuan M.; Sinatra L.; Alyami N. M.; Liu J.; Yassitepe E.; Yang Z.; Voznyy O.; Comin R.; Hedhili M. N.; Mohammed O. F.; Lu Z. H.; Kim D. H.; Sargent E. H.; Bakr O. M. Highly efficient perovskite-quantum-dot light-emitting diodes by surface engineering. Adv. Mater. 2016, 28 (39), 8718–8725. 10.1002/adma.201600784. 27529532

[ref10] Otero-Martinez C.; Zaffalon M. L.; Ivanov Y. P.; Livakas N.; Goldoni L.; Divitini G.; Bora S.; Saleh G.; Meinardi F.; Fratelli A.; Chakraborty S.; Polavarapu L.; Brovelli S.; Manna L. Ultrasmall CsPbBr_3_ Blue Emissive Perovskite Quantum Dots Using K-Alloyed Cs_4_PbBr_6_ Nanocrystals as Precursors. ACS Energy Letters 2024, 9 (5), 2367–2377. 10.1021/acsenergylett.4c00693. 39372427 PMC11450558

[ref11] De Trizio L.; Infante I.; Manna L. Surface chemistry of lead halide perovskite colloidal nanocrystals. Acc. Chem. Res. 2023, 56 (13), 1815–1825. 10.1021/acs.accounts.3c00174. 37347953 PMC10324302

[ref12] Yang D.; Li X.; Zeng H. Surface chemistry of all inorganic halide perovskite nanocrystals: passivation mechanism and stability. Advanced Materials Interfaces 2018, 5 (8), 1701662 10.1002/admi.201701662.

[ref13] De Roo J.; Ibáñez M.; Geiregat P.; Nedelcu G.; Walravens W.; Maes J.; Martins J. C.; Van Driessche I.; Kovalenko M. V.; Hens Z. Highly dynamic ligand binding and light absorption coefficient of cesium lead bromide perovskite nanocrystals. ACS Nano 2016, 10 (2), 2071–2081. 10.1021/acsnano.5b06295. 26786064

[ref14] Huang H.; Raith J.; Kershaw S. V.; Kalytchuk S.; Tomanec O.; Jing L.; Susha A. S.; Zboril R.; Rogach A. L. Growth mechanism of strongly emitting CH_3_NH_3_PbBr_3_ perovskite nanocrystals with a tunable bandgap. Nat. Commun. 2017, 8 (1), 996 10.1038/s41467-017-00929-2. 29042559 PMC5715004

[ref15] Akkerman Q. A.; D’innocenzo V.; Accornero S.; Scarpellini A.; Petrozza A.; Prato M.; Manna L. Tuning the optical properties of cesium lead halide perovskite nanocrystals by anion exchange reactions. J. Am. Chem. Soc. 2015, 137 (32), 10276–10281. 10.1021/jacs.5b05602. 26214734 PMC4543997

[ref16] Imran M.; Ijaz P.; Goldoni L.; Maggioni D.; Petralanda U.; Prato M.; Almeida G.; Infante I.; Manna L. Simultaneous cationic and anionic ligand exchange for colloidally stable CsPbBr_3_ nanocrystals. ACS Energy Letters 2019, 4 (4), 819–824. 10.1021/acsenergylett.9b00140.

[ref17] Zhang B.; Goldoni L.; Zito J.; Dang Z.; Almeida G.; Zaccaria F.; De Wit J.; Infante I.; De Trizio L.; Manna L. Alkyl phosphonic acids deliver CsPbBr_3_ nanocrystals with high photoluminescence quantum yield and truncated octahedron shape. Chem. Mater. 2019, 31 (21), 9140–9147. 10.1021/acs.chemmater.9b03529.

[ref18] Wang Q.; Zheng X.; Deng Y.; Zhao J.; Chen Z.; Huang J. Stabilizing the α-phase of CsPbI_3_ perovskite by sulfobetaine zwitterions in one-step spin-coating films. Joule 2017, 1 (2), 371–382. 10.1016/j.joule.2017.07.017.

[ref19] Almeida G.; Ashton O. J.; Goldoni L.; Maggioni D.; Petralanda U.; Mishra N.; Akkerman Q. A.; Infante I.; Snaith H. J.; Manna L. The phosphine oxide route toward lead halide perovskite nanocrystals. J. Am. Chem. Soc. 2018, 140 (44), 14878–14886. 10.1021/jacs.8b08978. 30358392 PMC6438589

[ref20] Morad V.; Stelmakh A.; Svyrydenko M.; Feld L. G.; Boehme S. C.; Aebli M.; Affolter J.; Kaul C. J.; Schrenker N. J.; Bals S.; Sahin Y.; Dirin D. N.; Cherniukh I.; Raino G.; Baumketner A.; Kovalenko M. V. Designer phospholipid capping ligands for soft metal halide nanocrystals. Nature 2024, 626 (7999), 542–548. 10.1038/s41586-023-06932-6. 38109940 PMC10866715

[ref21] Krieg F.; Ochsenbein S. T.; Yakunin S.; Ten Brinck S.; Aellen P.; Süess A.; Clerc B.; Guggisberg D.; Nazarenko O.; Shynkarenko Y.; Kumar S.; Shih C.-J.; Infante I.; Kovalenko M. V. Colloidal CsPbX_3_ (X= Cl, Br, I) nanocrystals 2.0: Zwitterionic capping ligands for improved durability and stability. ACS Energy Letters 2018, 3 (3), 641–646. 10.1021/acsenergylett.8b00035. 29552638 PMC5848145

[ref22] Zhang Y.; Hou G.; Wu Y.; Chen M.; Dai Y.; Liu S.; Zhao Q.; Lin H.; Fang J.; Jing C.; Chu J. Surface reconstruction of CsPbBr_3_ nanocrystals by the ligand engineering approach for achieving high quantum yield and improved stability. Langmuir 2023, 39 (17), 6222–6230. 10.1021/acs.langmuir.3c00393. 37079335

[ref23] Cai Y.; Li W.; Tian D.; Shi S.; Chen X.; Gao P.; Xie R. J. Organic Sulfonium-Stabilized High-Efficiency Cesium or Methylammonium Lead Bromide Perovskite Nanocrystals. Angew. Chem., Int. Ed. 2022, 61 (37), e202209880 10.1002/anie.202209880. 35852816

[ref24] Liu H.; Shonde T. B.; Olasupo O. J.; Islam M. S.; Manny T. F.; Woodhouse M.; Lin X.; Vellore Winfred J. R.; Mao K. S.; Lochner E.; Fatima I.; Hanson K.; Ma B. Organic semiconducting ligands passivated CsPbBr_3_ nanoplatelets for blue light-emitting diodes. ACS Energy Letters 2023, 8 (10), 4259–4266. 10.1021/acsenergylett.3c01576.

[ref25] Xu L.-J.; Worku M.; Lin H.; Xu Z.; He Q.; Zhou C.; Zhang H.; Xin Y.; Lteif S.; Xue J.; Ma B. Highly Emissive and stable organic-perovskite nanocomposite thin films with phosphonium passivation. J. Phys. Chem. Lett. 2019, 10 (19), 5923–5928. 10.1021/acs.jpclett.9b02387. 31529944

[ref26] Yoon Y. J.; Lee K. T.; Lee T. K.; Kim S. H.; Shin Y. S.; Walker B.; Park S. Y.; Heo J.; Lee J.; Kwak S. K.; Kim G.-H.; Kim J. Y. Reversible, full-color luminescence by post-treatment of perovskite nanocrystals. Joule 2018, 2 (10), 2105–2116. 10.1016/j.joule.2018.07.012.

[ref27] Gamarra A.; Urpi L.; Martinez de Ilarduya A.; Munoz-Guerra S. Crystalline structure and thermotropic behavior of alkyltrimethylphosphonium amphiphiles. Phys. Chem. Chem. Phys. 2017, 19 (6), 4370–4382. 10.1039/C6CP08304H. 28119974

[ref28] Shynkarenko Y.; Bodnarchuk M. I.; Bernasconi C.; Berezovska Y.; Verteletskyi V.; Ochsenbein S. T.; Kovalenko M. V. Direct synthesis of quaternary alkylammonium-capped perovskite nanocrystals for efficient blue and green light-emitting diodes. ACS Energy Letters 2019, 4 (11), 2703–2711. 10.1021/acsenergylett.9b01915. 31737780 PMC6849336

[ref29] Zaccaria F.; Zhang B.; Goldoni L.; Imran M.; Zito J.; van Beek B.; Lauciello S.; De Trizio L.; Manna L.; Infante I. The Reactivity of CsPbBr_3_ Nanocrystals toward Acid/Base Ligands. ACS Nano 2022, 16 (1), 1444–1455. 10.1021/acsnano.1c09603. 35005882 PMC8793808

[ref30] Liu J.; Song K.; Shin Y.; Liu X.; Chen J.; Yao K. X.; Pan J.; Yang C.; Yin J.; Xu L.-J.; Yang H.; El-Zohry A. M.; Xin B.; Mitra S.; Hedhili M. N.; Roqan I. S.; Mohammed O. F.; Han Y.; Bakr O. M. Light-induced self-assembly of cubic CsPbBr_3_ perovskite nanocrystals into nanowires. Chem. Mater. 2019, 31 (17), 6642–6649. 10.1021/acs.chemmater.9b00680.

[ref31] Wider G.; Dreier L. Measuring protein concentrations by NMR spectroscopy. J. Am. Chem. Soc. 2006, 128 (8), 2571–2576. 10.1021/ja055336t. 16492040

[ref32] Otero-Martínez C.; Ye J.; De Trizio L.; Goldoni L.; Rao A.; Pérez-Juste J.; Hoye R. L.; Manna L.; Polavarapu L. Organic A-Site Cations Improve the Resilience of Inorganic Lead-Halide Perovskite Nanocrystals to Surface Defect Formation. Adv. Funct. Mater. 2024, 34, 2404399 10.1002/adfm.202404399.

[ref33] Diroll B. T.; Nedelcu G.; Kovalenko M. V.; Schaller R. D. High-temperature photoluminescence of CsPbX_3_ (X = Cl, Br, I) nanocrystals. Adv. Funct. Mater. 2017, 27 (21), 1606750 10.1002/adfm.201606750.

[ref34] Ijaz P.; Imran M.; Soares M. M.; Tolentino H. l. C.; Martín-García B.; Giannini C.; Moreels I.; Manna L.; Krahne R. Composition-, size-, and surface functionalization-dependent optical properties of lead bromide perovskite nanocrystals. J. Phys. Chem. Lett. 2020, 11 (6), 2079–2085. 10.1021/acs.jpclett.0c00266. 32090576 PMC7997568

[ref35] Dar M. I.; Jacopin G.; Meloni S.; Mattoni A.; Arora N.; Boziki A.; Zakeeruddin S. M.; Rothlisberger U.; Grätzel M. Origin of unusual bandgap shift and dual emission in organic-inorganic lead halide perovskites. Science Advances 2016, 2 (10), e1601156 10.1126/sciadv.1601156. 27819049 PMC5091363

[ref36] Sercel P. C.; Lyons J. L.; Wickramaratne D.; Vaxenburg R.; Bernstein N.; Efros A. L. Exciton fine structure in perovskite nanocrystals. Nano Lett. 2019, 19 (6), 4068–4077. 10.1021/acs.nanolett.9b01467. 31088061

[ref37] Filippi U.; Toso S.; Zaffalon M. L.; Pianetti A.; Li Z.; Marras S.; Goldoni L.; Meinardi F.; Brovelli S.; Baranov D.; Manna L. Cooling-Induced Order-Disorder Phase Transition in CsPbBr_3_ Nanocrystal Superlattices. Adv. Mater. 2025, 37 (3), 2410949 10.1002/adma.202410949. 39568247 PMC11756043

[ref38] Pan J.; Shang Y.; Yin J.; De Bastiani M.; Peng W.; Dursun I.; Sinatra L.; El-Zohry A. M.; Hedhili M. N.; Emwas A.-H.; Mohammed O. F.; Ning Z.; Bakr O. M. Bidentate ligand-passivated CsPbI_3_ perovskite nanocrystals for stable near-unity photoluminescence quantum yield and efficient red light-emitting diodes. J. Am. Chem. Soc. 2018, 140 (2), 562–565. 10.1021/jacs.7b10647. 29249159

[ref39] Imran M.; Ijaz P.; Baranov D.; Goldoni L.; Petralanda U.; Akkerman Q.; Abdelhady A. L.; Prato M.; Bianchini P.; Infante I.; Manna L. Shape-pure, nearly monodispersed CsPbBr_3_ nanocubes prepared using secondary aliphatic amines. Nano Lett. 2018, 18 (12), 7822–7831. 10.1021/acs.nanolett.8b03598. 30383965 PMC6428374

[ref40] Dai J.; Zhao C.; Xu J.; Roshan H.; Dong H.; Di Stasio F.; Yuan F.; Jiao B.; Wu Z. Double hole transport layers deliver promising-performance in light-emitting diodes based on MAPbBr_3_ nanocrystals. Org. Electron. 2024, 124, 106941 10.1016/j.orgel.2023.106941.

[ref41] Dai J.; Roshan H.; De Franco M.; Goldoni L.; De Boni F.; Xi J.; Yuan F.; Dong H.; Wu Z.; Di Stasio F.; Manna L. Partial Ligand Stripping from CsPbBr_3_ Nanocrystals Improves Their Performance in Light-Emitting Diodes. ACS Appl. Mater. Interfaces 2024, 16 (9), 11627–11636. 10.1021/acsami.3c15201. 38381521 PMC11932522

[ref42] Kim J. S.; Heo J.-M.; Park G.-S.; Woo S.-J.; Cho C.; Yun H. J.; Kim D.-H.; Park J.; Lee S.-C.; Park S.-H.; Yoon E.; Greenham N. C.; Lee T.-W. Ultra-bright, efficient and stable perovskite light-emitting diodes. Nature 2022, 611 (7937), 688–694. 10.1038/s41586-022-05304-w. 36352223

[ref43] Zheng W.; Wan Q.; Liu M.; Zhang Q.; Zhang C.; Yan R.; Feng X.; Kong L.; Li L. CsPbBr_3_ nanocrystal light-emitting diodes with efficiency up to 13.4% achieved by careful surface engineering and device engineering. J. Phys. Chem. C 2021, 125 (5), 3110–3118. 10.1021/acs.jpcc.0c11085.

[ref44] Gutiérrez Álvarez S.; Lin W.; Abdellah M.; Meng J.; Zidek K.; Pullerits T. n.; Zheng K. Charge carrier diffusion dynamics in multisized quaternary alkylammonium-capped CsPbBr_3_ perovskite nanocrystal solids. ACS Appl. Mater. Interfaces 2021, 13 (37), 44742–44750. 10.1021/acsami.1c11676. 34515458 PMC8461607

[ref45] Kong L.; Luo Y.; Wu Q.; Xiao X.; Wang Y.; Chen G.; Zhang J.; Wang K.; Choy W. C.; Zhao Y.-B.; Li H.; Chiba T.; Kido J.; Yang X. Efficient and stable hybrid perovskite-organic light-emitting diodes with external quantum efficiency exceeding 40%. Light: Science & Applications 2024, 13 (1), 138 10.1038/s41377-024-01500-7. PMC11169476 38866757

[ref46] Qian X.-Y.; Tang Y.-Y.; Zhou W.; Shen Y.; Guo M.-L.; Li Y.-Q.; Tang J.-X. Strategies to improve luminescence efficiency and stability of blue perovskite light-emitting devices. Small Science 2021, 1 (8), 2000048 10.1002/smsc.202000048. 40213163 PMC11935876

