# Supplementary material for: Improving the Stability of Colloidal CsPbBr3 Nanocrystals with an Alkylphosphonium Bromide as Surface Ligand Pair
Source: ACS Energy Lett. 2025 Apr 11;10(5):2268–76. doi: 10.1021/acsenergylett.5c00124 (PMC12070459; doi:10.1021/acsenergylett.5c00124)
Supplement: Supplementary file 1 — nz5c00124_si_001.pdf [file nz5c00124_si_001.pdf]

Supporting Information for:

Improving the Stability of Colloidal $CsPbBr_3$ Nanocrystals with an Alkylphosphonium Bromide as Surface Ligand Pair

Meenakshi Pegu[1,#], Hossein Roshan[2,#], Clara Otero-Martínez[1], Luca Goldoni[3], Juliette Zito[1], Nikolaos Livakas[1,4], Pascal Rusch[1], Francesco De Boni[3], Francesco Di Stasio[2], Ivan Infante,[5,6] Luca De Trizio[7*], Liberato Manna[1]*

[1] Nanochemistry, [2] Photonic Nanomaterials, [3] Materials Characterization, [7] Chemistry Facility, Istituto Italiano di Tecnologia, Via Morego 30, 16163 Genova, Italy

[4] Dipartimento di Chimica e Chimica Industriale, Università di Genova,16146 Genova, Italy

[5] BCMaterials, Basque Center for Materials, Applications, and Nanostructures, UPV/EHU Science Park, Leioa 48940, Spain

[6] Ikerbasque Basque Foundation for Science, Bilbao 48009, Spain

Table S1. List of phosphorous-based ligands used for perovskite NCs synthesis.

Ligand	Perovskite NCs	Year	Efficiency	References
Trioctylphosphine	$CsPbBr_3$	2018	PLQY = 50 %<br>EQE = 0.014 %	*Joule* 2.10 (2018): 2105-2116.
Ethane-1,2-diylbis(triphenyl phosphonium) bromide	$CsPbBr_3$	2019	PLQE = 78 %<br>EQE = 6.3 %	*The Journal of Physical Chemistry Letters* 10.19 (2019): 5923-5928.
Triphenyl(9-phenyl-9H-carbazol-3-yl) phosphonium bromide	$CsPbBr_3$	2019	PLQE = 77.6 %	*The Journal of Physical Chemistry Letters* 10.19 (2019): 5836-5840.
Phenylphosphonic dichloride	$CsPbCl_3$	2021	PLQY = 71 %<br>EQE = 0.18 %	*ACS Energy Letters* 6.10 (2021): 3545-3554.
Trihexyl(tetradecyl)phosphonium bromide	$CsPbBr_3$	2023	PLQY = 55 %	*The Journal of Chemical Physics* 158.17 (2023).
Triphenyl (9-phenyl-9H-carbazol-3-yl) phosphonium bromide	$CsPbBr_3$	2023	EQE = 4.15 %	*ACS Energy Letters* 8.10 (2023): 4259-4266.

Experimental Section

Materials.

Cesium carbonate (Cs_2CO_3, reagent plus, 99 %), lead acetate trihydrate ($PbAc_2$. $3H_2O$, 99.99 %), oleic acid (OA, 90 %), benzoyl bromide (C_6H_5OBr, 97 %), octadecene (ODE, technical grade, 90%), toluene (anhydrous, 99.5 %), ethyl acetate (anhydrous, 99.8 %), trimethyl phosphine (PMe_3, 97%), 1-bromotetradecane (97 %), diethyl ether (HPLC grade, 99.9%) dimethyl sulfone (DMS, TraceCERT Certified Reference Materials for quantitative NMR, 99.99%), toluene d8 (99 atom % D), chloroform-d ($CDCl_3$, 99.8 atom % D), 1,4-dioxane (anhydrous, 99.5 %), chlorobenzene (anhydrous, 99.5 %), dimethylsufoxide-d6 (DMSO-d6, 99.9 atom % D), lithium fluoride (LiF, 99.9%), and Polyvinyl Carbazole (PVK) are purchased from Sigma Aldrich. Didodecylamine (DDAm, 97 %) is purchased from TCI. Poly(3,4-ethylenedioxythiophene):poly(styrene sulfonate) (PEDOT:PSS) with ratio of 1:6 (AI 4083) was purchased from Ossila. Patterned indium tin oxide (ITO) substrates, Poly(triaryl amine) (PTAA), and 2′,2′-(1,3,5-benzinetriyl)-tris(1-phenyl-1-H-benzimidazole) (TPBi, 98%) purchased from Lumtech. Aluminum pallets were purchased from Ted Pella for evaporation use. Unless otherwise stated, all the materials are used without further purification.

General procedure for the synthesis.

Synthesis of trimethyl(tetradecyl)phosphonium bromide or TTP-Br ligand.

To an oven-dried three-neck Schlenk round bottom flask equipped with a magnetic stir bar 1-Bromotetradecane (5.5 mmol) in anhydrous toluene (10 mL) is added and degassed for 5 minutes at room temperature. 5 mL of trimethyl phosphine (5 mmol) is added slowly under a nitrogen atmosphere, after which the mixture is slowly heated to 80°C and then stirred for 18 hours under reflux conditions. After reaction completion, the mixture is concentrated under reduced pressure to obtain a crude solid. This solid is further washed with diethyl ether (5 times x 20 mL) using a Buchner funnel and dried under vacuum at room temperature, to obtain the final product as TTP-Br. White solid obtained 1.32 g, yield 75 %. The chemical structure and purity of the TTP-Br ligand were ascertained by ^1H NMR and ^{13}C NMR in toluene d8 (see Supporting Information for peak assignment, **Figures S1-S3**).

Scheme S1. Synthesis route for trimethyl(tetradecyl)phosphonium bromide (TTP-Br).

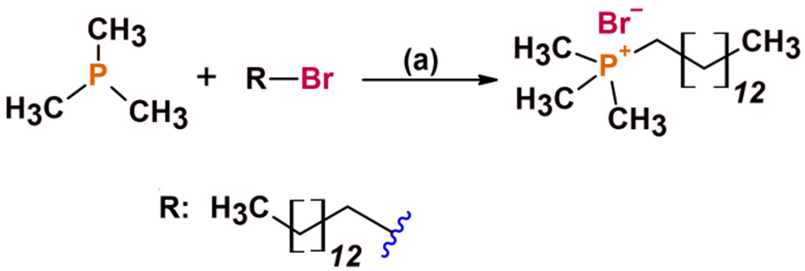

Reaction condition: (a) 10 mL toluene, 80°C, 18h reflux.

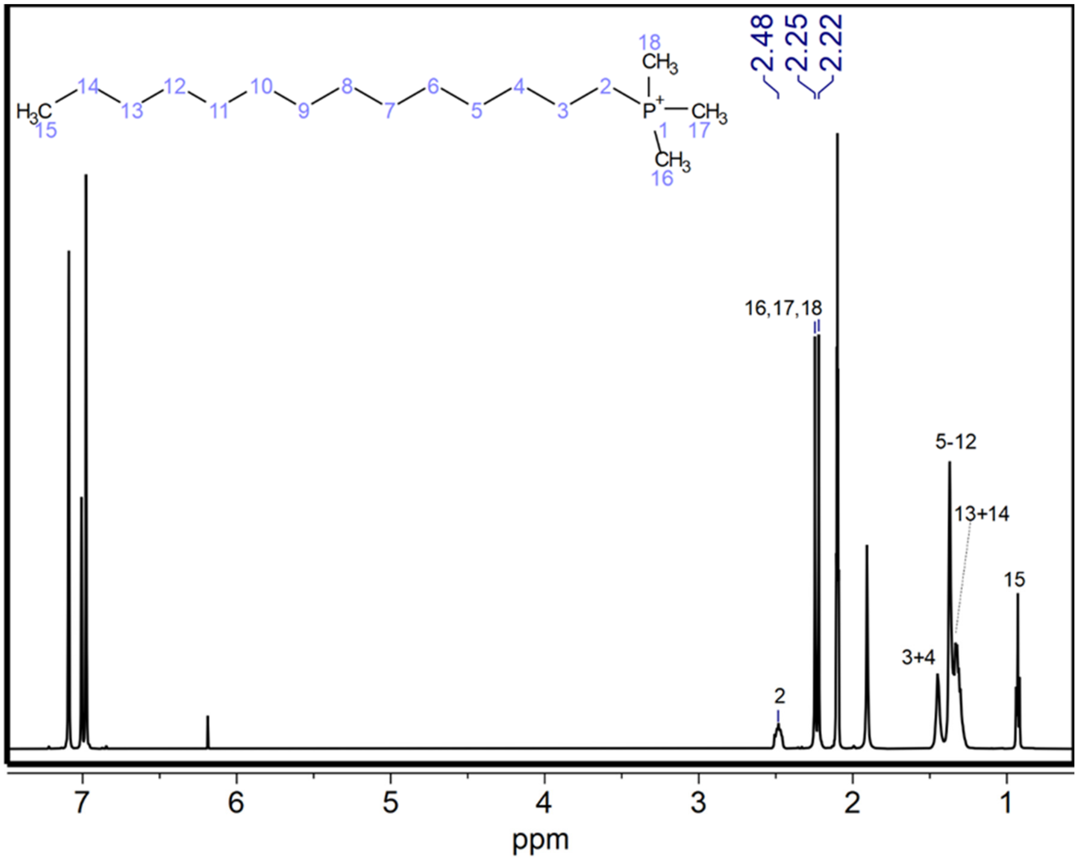

Figure S1. ^{1}H NMR spectrum of TTP-Br in toluene-D at 298 K, with peak assignment, the TTP-Br structure formula, and signal numbering.

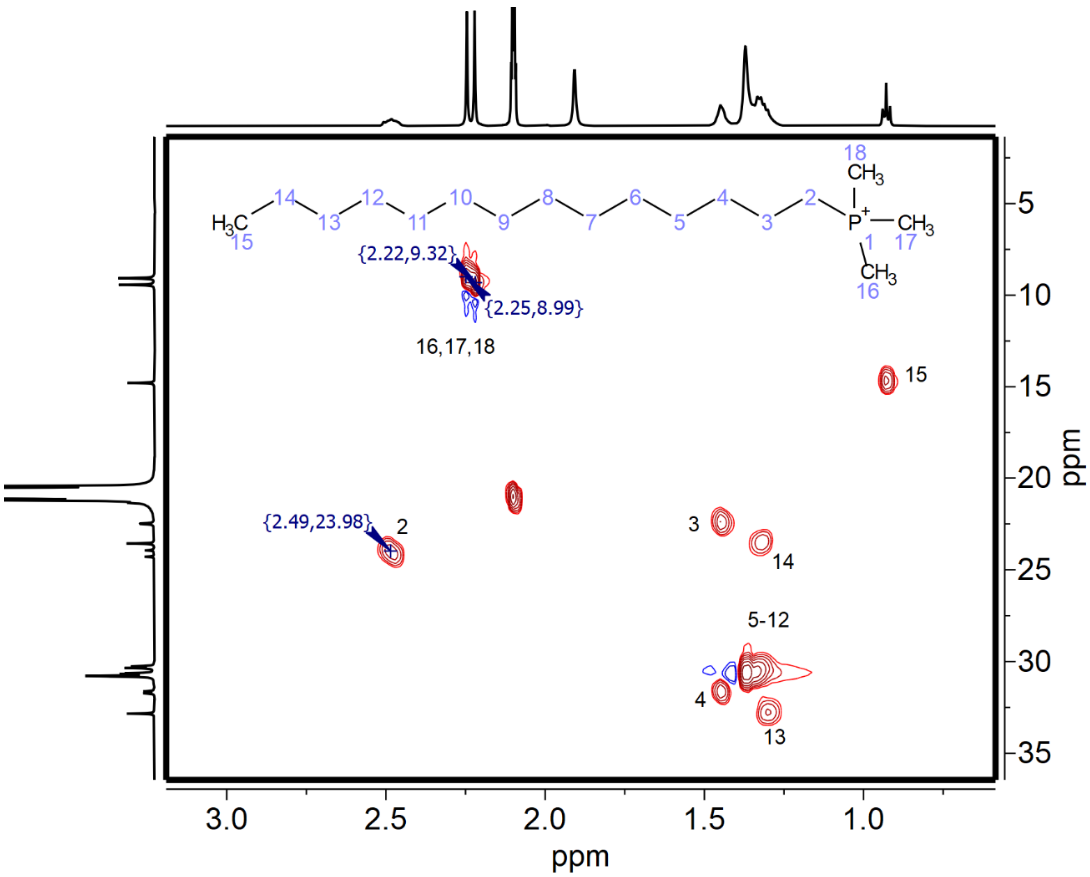

Figure S2. ^{1}H-^{13}C HSQC NMR spectrum of TTP-Br in toluene-D at 298K, with peak assignment, the TTP-Br structure formula, and signal numbering.

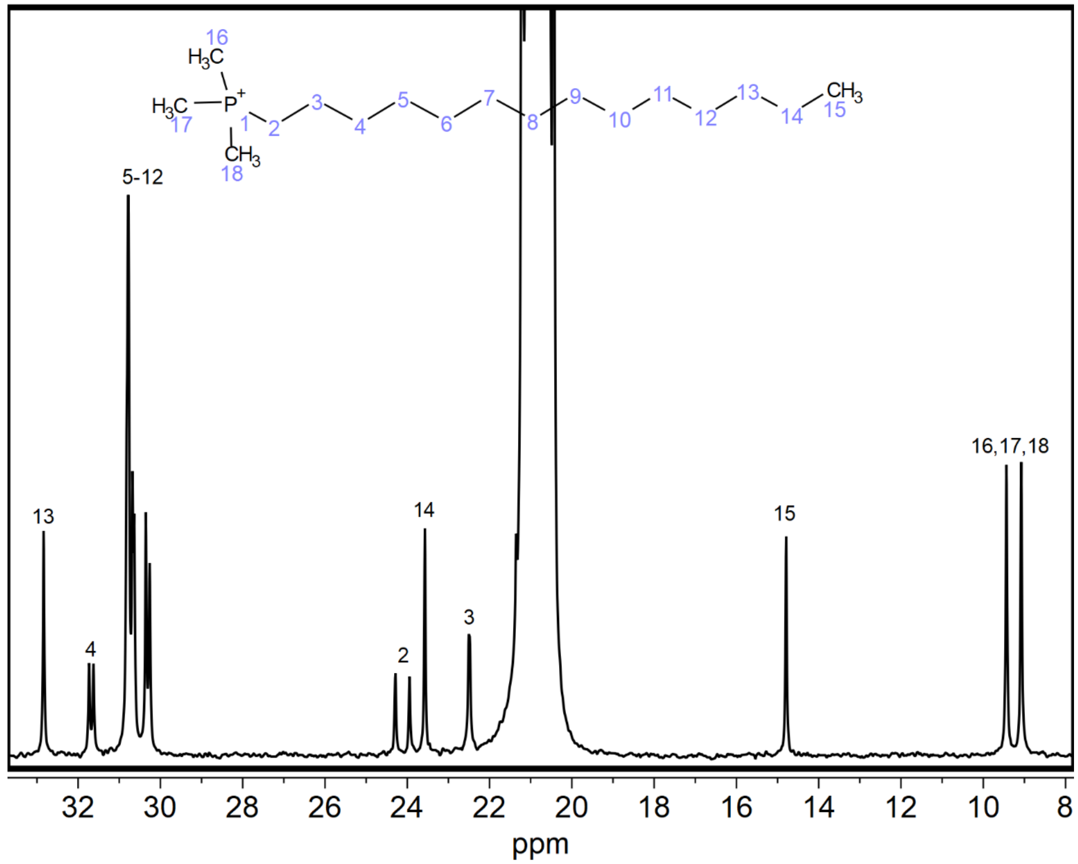

Figure S3. ^{13}C {^1H} decoupled NMR spectrum of TTP-Br in toluene-D at 298K, with peak assignment, the TTP-Br structure formula, and signal numbering.

Preparation of Cs-Pb-Oleate stock solution.

$Pb(CH_3COO)_2 \cdot 3\ H_2O$ (760 mg), Cs_2CO_3 (160 mg), and oleic acid (15 mL) are loaded in a 25 mL three-neck flask. The mixture is heated up to 100°C and degassed using a Schlenk line for 1.5 hours. At this point, the precursor salt is completely dissolved, and the solution is cooled down to room temperature and stored in a glove box.

Preparation of Didodecylamine stock solution.

In a glovebox, 10 mL of anhydrous toluene is added to 4.43 g of Didodecylamine (DDA) in a 25 mL vial. The mixture is then placed on a hot plate and heated to 60°C for 10 minutes until the DDA in the mixture is completely dissolved. After cooling, the mixture is used for the subsequent synthesis.

Synthesis of $CsPbBr_3$ NCs.

The Cs-Oleate-capped $CsPbBr_3$ NCs are synthesized following the protocol of Imran et al. reported using the standard Schlenk technique.[1, 2] Firstly, 1.5 mL of Cs-Pb-Oleate stock solution, 1.5 mL of Didodecylamine (DDA) stock solution, and 9 mL of 1-octadecene are loaded together into a 40 mL glass vial. The mixture is heated up to 90°C and degassed for 2 hours. It is then heated up to 100°C under nitrogen, and at that point, a solution of benzoyl bromide (50 µL) in anhydrous toluene (500 µL) is injected swiftly. After 60 seconds, the reaction is quenched by cooling in an ice-water bath and directly used for ligand exchange. The crude Cs-Oleate-capped $CsPbBr_3$ NCs solution is divided into three different fractions (3 mL each); one fraction is used as the reference, and the other two fractions are used for post-treatment ligand exchange. The organic solvents used in the present work were anhydrous to avoid moisture contamination. All the samples have been cleaned to remove the excess ligands prior to their structural and optical characterizations.

Ligand exchange and washing of NCs.

Each of the three fractions (3 mL) of crude Cs-Oleate-capped $CsPbBr_3$ NCs solution is used for the post-synthesis ligand exchange treatment with 2 mL DDA-Br (25 mM), and 2 mL TTP-Br (25 mM), respectively. TTP-Br halide salts have lower solubility in nonpolar solvents such as toluene. In this case, we added a small amount of chloroform (0.5 mL) to dissolve TTP-Br in toluene (4.5 mL) and heated at 80ºC for five minutes. The complete solubility of TTP-Br maximized the passivating effect on the perovskite NCs' surface. A similar exchange protocol was followed for the DDA-Br-capped NCs. The vials containing the mixtures of DDA-Br-capped and TTP-Br-capped NCs are then stirred for 20 mins under a nitrogen atmosphere. After that, the NCs are precipitated and washed, adding an excess of anhydrous ethyl acetate (20 mL), and separated upon centrifugation at 6000 rpm. The supernatant is discarded to remove the unreacted residues and ligands from the precipitate and is redispersed in anhydrous toluene (1 mL). The washing process was repeated by adding ethyl acetate (6 mL) for the second time, centrifuged at 6000 rpm, and finally redispersed in neat toluene (1mL).

The overall washing procedure was carried out two times following the same procedure with pure anhydrous toluene only (without using ligands) for the crude Cs-Oleate-capped $CsPbBr_3$ NCs. Organic solvents used in the present work are anhydrous to avoid moisture contamination.

We have observed that further addition of TTP-Br ligand after the second wash resulted in the degradation of the NCs, adversely affecting both their size and morphology (**Figure S42**).

LED Fabrication using TTP-Br-capped NCs.

First, the patterned ITO glass substrate was ultrasonically cleaned sequentially using an ITO cleaning solution, acetone, and isopropanol, then dried with a nitrogen flow. Subsequently, substrates underwent ultraviolet-ozone treatment to achieve a hydrophilic surface. For the LED devices, a PEDOT:PSS solution was spin-coated onto the treated ITO glass at 5000 rpm for 40 seconds and then annealed at 120°C for 10 minutes under ambient conditions. The ITO/PEDOT:PSS substrates were then transferred to a glovebox where a single-hole transport layer of PTAA (10 mg/mL in chlorobenzene) or PVK (3 mg/mL in 1,4-dioxane) was spin-coated at 2000 rpm for 45 seconds and annealed at 75°C for 10 minutes. For a double-hole transport layer, PTAA (10 mg/mL) was first spin-coated and annealed at 75°C for 10 minutes. Once cooled, an upper PVK layer (3 mg/mL) was applied using dynamic spin coating. The

emitting layer (TTP-Br-capped $CsPbBr_3$ NCs) was formed by dynamic spin-coating of the NCs solution at 2000 rpm for 45 seconds. Electron transport layers of TPBi (35 nm), LiF (1 nm), and an aluminum electrode (100 nm) were sequentially deposited in a vacuum chamber through a shadow mask at a base pressure of 2×10^{-6} Torr. The thickness of the deposited layers was monitored using a quartz crystal. The active device area, defined by the overlap between the ITO and aluminum electrodes, was approximately 4 mm^2.

LED Characterization.

The JVL properties were assessed utilizing a Keithley 2636 source-measure unit in conjunction with a Thorlabs PDA 100 A silicon switchable gain detector. The silicon detector's output was transformed into power (photon flux) based on its responsivity. All measurements were done in the air with almost 50% of relative humidity. For luminance measurements, the surface of ITO/glass was considered as the light output without using an integrated sphere. To determine the EQE, we calculated the ratio of photon flux to the device's driving current. EL spectra were gathered using an Edinburgh Instrument FLS 900 spectrometer step scan.

Methods.

Powder X-ray diffraction (XRD) analysis.

XRD patterns are acquired using the PANanalytical Empyrean X-ray diffractometer equipped with a 1.8 kW Cu Kα ceramic X-ray tube and a PIXcel3D 2x2 area detector, operating at 40 mA and 45 kV using parallel beam geometry and symmetric reflection mode. The NC samples for diffraction measurements are prepared by concentrating the NC solution under nitrogen and drop-casting the dispersions on a zero-diffraction Si substrate. XRD patterns were acquired at room temperature under ambient conditions.

Transmission Electron Microscopy (TEM).

Conventional bright-field TEM images are acquired on samples prepared by drop casting the diluted NC colloids onto 200 mesh carbon film-coated copper grids using a JEOL-JEM1400Plus transmission electron microscope operating at an acceleration voltage of 120 kV. Scanning TEM coupled with energy-dispersive X-ray spectroscopy (EDS) was performed on

samples prepared in the same way and which were stored under dynamic vacuum conditions for 24 hours, using an image-aberration corrected JEOL JEM2200FS microscope with Schottky emitter operated at 200 kV and a Bruker X-Flash 5060 silicon-drift detector with 60 mm^2 area.

Optical Absorption and Photoluminescence Spectroscopy.

Optical absorption and photoluminescence spectra were recorded using a Varian Cary 300 UV-Vis spectrophotometer and a Varian Cary Eclipse spectrofluorometer using an excitation wavelength of 350 nm for all the samples. The samples are prepared by diluting NC colloids dispersed in toluene in quartz cuvettes with a 1 cm path length.

Photoluminescence Quantum Yield (PLQY) and Time-Correlated Single-Photon Counting (TCSPC).

The PLQY of the NC samples is measured using an FS5 Spectrofluorometer-Edinburgh Instruments, equipped with the integrating sphere with step increments of 0.5 nm and an integration time of 0.2 s per data point for one scan. The NC samples are prepared by diluting 10-15 μL of the concentrated NC solutions in 3 mL of anhydrous toluene within quartz cuvettes (1 cm path length), capped with white PTFE stoppers (Helma-Analytics, part number 111-10-40). A blank reference sample is prepared using 3 mL of anhydrous toluene. All the NC solutions are then diluted to obtain an optical density of ~ 0.20 - 0.21 at 375 nm excitation wavelength and measured with the integrating sphere using the output of the continuous Xenon lamp.

Time-resolved photoluminescence spectra (TRPL) at room temperature are obtained using an FLS900 Edinburgh spectrophotometer, and the PL decay traces are measured with a pulsed laser diode (λex = 375 nm, 10 MHz repetition rate, 60 ps pulse width) and fitted with a three-exponential decay function.

X-ray Photoluminescence Spectroscopy (XPS).

X-ray photoelectron spectroscopy (XPS) measurements were carried out through a Kratos Axis Ultra^{DLD} spectrometer (Kratos Analytical Ltd.) with a monochromated Al K_α X-ray source (*hv* = 1486.6 eV) operating at 20 mA and 15 kV. Specimens were prepared by dropping a concentrated NCs solution in toluene onto a highly ordered pyrolytic graphite (HOPG, ZYA

grade) substrate. The wide scans were collected over an analysis area of 300 × 700 μm^2 at a photoelectron pass energy of 160 eV and energy step of 1 eV, while high-resolution spectra of Cs 3d, Pb 4f, Br 3d, and C 1s, are collected at a photoelectron pass energy of 20 eV and an energy step of 0.1 eV. A take-off angle of 0° concerning the sample's normal direction was used for all analyses. The slight differential electrical charging effects (less than 0.5 eV) observed on all samples were not neutralized. The irradiation of an $APbX_3$-like perovskite with an electron beam is known to induce the desorption of halogen species and the nucleation of metallic Pb particles.[3] The spectra have been referenced to the adventitious carbon 1s peak at 284.8 eV. The spectra were analyzed with the Casa XPS software (Casa Software Ltd., version 2.3.24),[4] and the residual background was eliminated by the Shirley method across the binding energy range of the peaks of interest. The relative atomic concentrations were then estimated using the specific function in the Casa XPS software.

Ultraviolet photoelectron spectroscopy (UPS)

The ultraviolet photoelectron spectroscopy (UPS) measurements were performed using a He I (21.22 eV) discharge lamp, fitted in the same chamber used for XPS analyses, on an area of 55 μm in diameter, at a pass energy of 10 eV and with a dwell time of 100 ms. The energy levels within the equipment and UPS-based calculations are described as follows. The work function, φ, i.e. the position of the Fermi level versus the vacuum level, was determined for each sample from the position of the secondary electron cutoff in the UPS spectrum, using the following equation: $\boldsymbol{\varphi = h\nu - E_0}$, where hν is the source energy (21.22 eV for He I photons) and E_0 is the secondary electron cut off. The position of the valence band maximum (VBM) versus the vacuum level, i.e. the ionization energy, E_{ion}, was determined from the width of the entire UPS spectrum, according to the following equation: $\boldsymbol{E_{ion} = h\nu - (E_0 - E_1) = h\nu - E_0 + E_1}$, where E_1 is the position of the VBM concerning the zero (Fermi) level.[5] The values E_0 and E_1 were determined in the UPS spectrum through the background functions "Edge Up" and "Edge Down", respectively, in the Casa XPS software. The error bar associated with this procedure was estimated to be equal to 0.2 eV.

Nuclear magnetic resonance (NMR).

NMR was performed on a Bruker Avance III 600 MHz (600.13 MHz) spectrometer, fit with a 5 mm QCI cryoprobe. The matching, tuning, and line shape resolution were adjusted and the 90° pulse was finally calibrated by an automatic pulse calculation routine, before acquisition.[6] The temperature was actively monitored on each sample tube and the sample was let to equilibrate 2 minutes inside the probe earlier than the pre-acquisition routines.

^{1}H NMR spectra were acquired at 298 K and 313 K, by accumulating 32 scans (64 for *q*-NMR and NCs) without steady ones, with inter-pulses delay of 30s (64 for *q*-NMR and NCs) and 65536 digit points, over a spectral width of 20.83 ppm with the offset positioned at 6.18 ppm.

Spectra were smoothed with an exponential function equivalent to 0.3 Hz before Fourier transform. The ligand concentration was determined in DMSO-D6 (Deuterated Dimethyl sulfoxide) by comparing the ligand signal integrated intensity with that of a 10 mM DMS standard solution freshly prepared, by using the PULCON (Pulse Length Based Concentration Determination) method.[7]

The ^{1}H–^{13}C HSQC (Heteronuclear Single Quantum Coherence) experiments were performed with 16 transients (64 for NCs), 2048 digit points, 256 increments and ^{1}J CH of 145 Hz, a spectral width of 15.15 ppm for ^{1}H and 165.8 ppm for ^{13}C, with a transmitter frequency offset positioned at 7.00 and 75.0 ppm, respectively.

The ^{1}H-^{1}H NOESY (Nuclear Overhauser Spectroscopy) experiments were acquired with 32 scans (64 for NCs and for NOESY at 313K), a mixing time of 300 ms, over a spectral width of 15.15 ppm, centered at 7.49 ppm.

^{13}C NMR spectra were acquired by using a 30° for the ^{13}C pulse excitation and a broadband decoupling for ^{1}H, with 18435 transients, a relaxation delay of 2s, over a spectral width of 236.65 ppm centered at 100.00 ppm.

Surface ligand density calculations.

The surface ligand density of the samples was estimated by calculating the ratio between the concentration of ligands to the NCs' surface obtained by NMR and ICP-OES, respectively. The concentrations of both ligand and Pb were obtained after evaporating the colloidal solution of the NCs in deuterated toluene (previously characterized). The resulting precipitate was thoroughly dried under a nitrogen flow and subsequently dissolved in the dried NCs in

deuterated-DMSO (200 µL). The NCs in the DMSO-d6 solution were then loaded into a SampleJet NMR tube of 3 mm for ligand quantification.

Ligand quantification. The concentration of different ligands (TTP-Br, DDA-Br, and Cs-Oleate) was quantified using *quantitative* NMR spectroscopy by comparing the integrated area of ligand signals, each normalized for the number of protons generating the peak (2H), to that of a solution of dimethyl sulfone 10 mM (TraceCERT®) used as a standard solution, in DMSO-d_6, the latter normalized to 6 H. The concentration of all the ligands was obtained by the integrated peak ratio between a proton signal which is the characteristic peak of each ligand, and that of the external standard solution through the PULCON (PUlse Length-based CONcentration determination) method[7]. In the case of TTP-Br, the signal employed was the multiplet at 2.14 ppm. For Cs-Oleate-capped NCs, the signal at 5.30 ppm which corresponds to the protons of the alkyl double bond of the oleate species was measured. Finally, the triplet at 3.20 ppm corresponding to the methyl group of the DDA-Br ligand was used to estimate its concentration.

Inductively Coupled Plasma−Optical Emission Spectroscopy (ICP-OES). After the ligand quantification on the deuterated DMSO solution, the Pb concentration of the NCs was estimated by ICP-OES on an aiCAP 6000 spectrometer (Thermo Scientific) Prior to the measurement, 50 µL of the DMSO – d_6 sample were diluted to 25 mL in an aqueous solution of aqua regia (1:25) and subjected to an acid digestion overnight.

Ligand density. The ligand density of the three samples was estimated by calculating the ratio of the ligand concentration and NC surface in the deuterated-DMSO solution:

$$Ligand\ density\ (ligand/nm^2) = \frac{[Ligand](mL^{-1})}{Total\ NC\ surface\ (nm^2/mL)}$$

The total NC surface is determined based on the Pb concentration in the DMSO-d_6 sample (measured by ICP analysis) and the size distribution of the NCs considering a unit cell size of 0.589 nm:

$$Total\ NC\ surface\ (nm^2/mL) = NC\ concentration\ (mL^{-1}) * [NC\ size\ (nm)]^2 * 6$$

$$NC\ concentration\ (mL^{-1}) = \frac{Total\ concetration\ of\ Pb\ (mL^{-1})}{Pb\ atoms/NC}$$

$$Pb\ atoms/NC = \left[\frac{NC\ size\ (nm)}{0.589\ nm/unit\ cell}\right]^3$$

Density functional theory (DFT) calculations.

We conducted atomistic simulations at the DFT level using the PBE exchange-correlation functional[8] and a double-ζ basis set with polarization functions[9], as implemented in CP2K version 6.1[10]. All structural optimizations were performed in a vacuum. Scalar relativistic effects were accounted for using effective core potential functions within the basis set. While spin-orbit coupling effects were not included, previous studies on similar systems have demonstrated that their influence on relaxed structural properties is negligible. Details regarding the model construction are provided in the main text as well as in the references[11, 12].

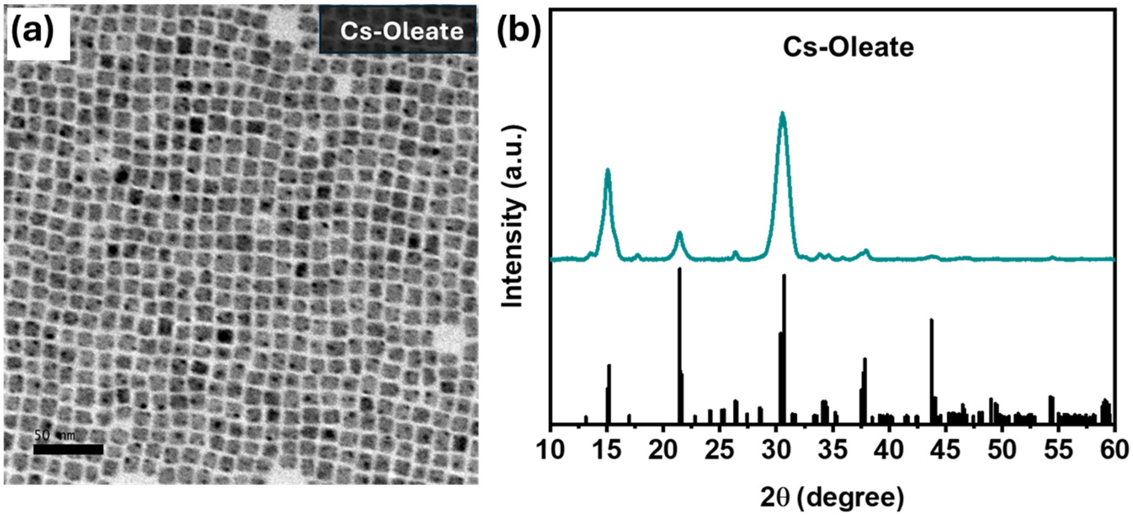

Figure S4. (a) TEM micrograph, and (b) XRD spectrum of Cs-Oleate-capped $CsPbBr_3$ NCs.

Table S2. Quantitative elemental analysis was obtained through XPS.

$CsPbBr_3$ NCs	Cs (%)	Pb (%)	Br (%)	Cs/Pb	Br/Pb	N (%)	O (%)	P (%)
**Cs-Oleate**	23.9	23.2	52.9	1.03	2.28	0	9.3	0
**DDA-Br**	22.5	20.5	57.0	1.10	2.78	2.7	2.5	0
**TTP-Br**	22.1	20.8	57.1	1.06	2.75	0	2.8	2.6

Elemental analysis using X-ray photoelectron spectroscopy (XPS) revealed the exchange of Cs-Oleate-capped $CsPbBr_3$ NCs with DDA-Br and TTP-Br ligands. A detailed explanation is provided in the manuscript. The observed reduction in the atomic percentage of Cs suggests the replacement of the Cs^+ with the PMe_3^+ group. Notably, N *1s* peaks appear only in DDA-Br-capped $CsPbBr_3$ NCs. The increase in Br/Pb ratio indicates halogen compensation during the treatment of the quaternary ammonium and phosphonium salts. The relative atomic percentages of the elements (Cs, Pb, Br, O, N, C, P) are presented in **Table S2**.

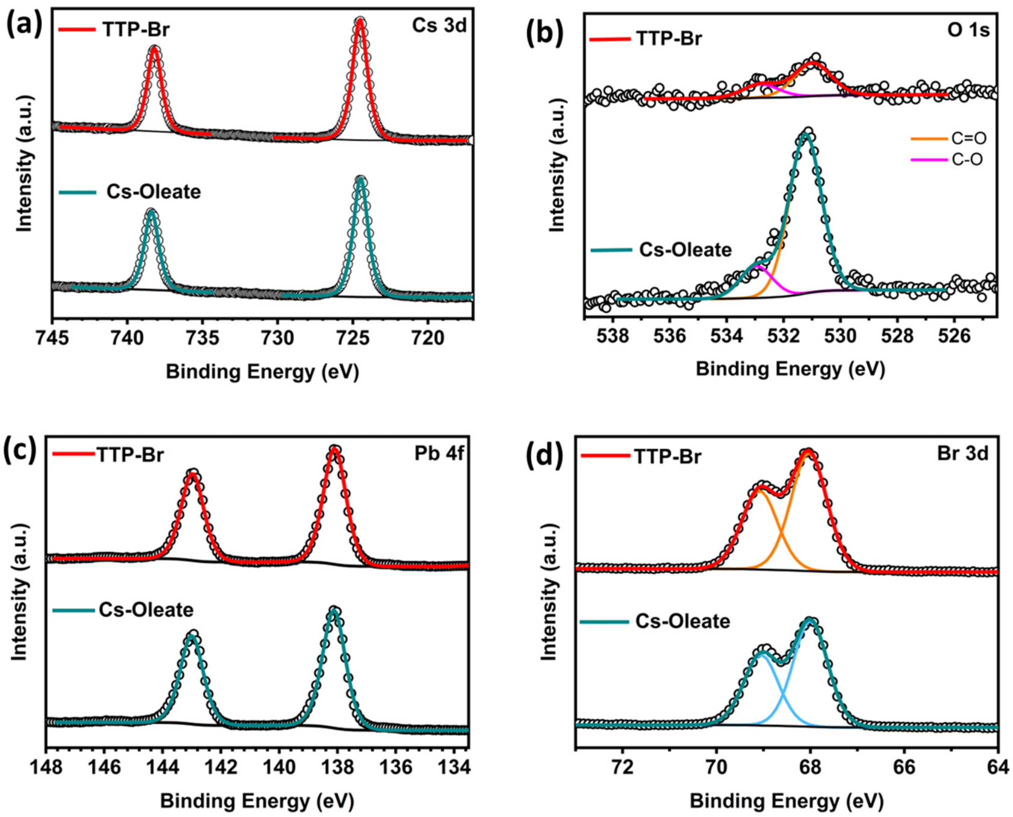

Figure S5. Comparison between (a) Cs 3*d*, (b) O 1*s*, (c) Pb 4*f*, (d) Br 3*d*, XPS spectra of TTP-Br-, and Cs-Oleate-capped $CsPbBr_3$ NCs.

.

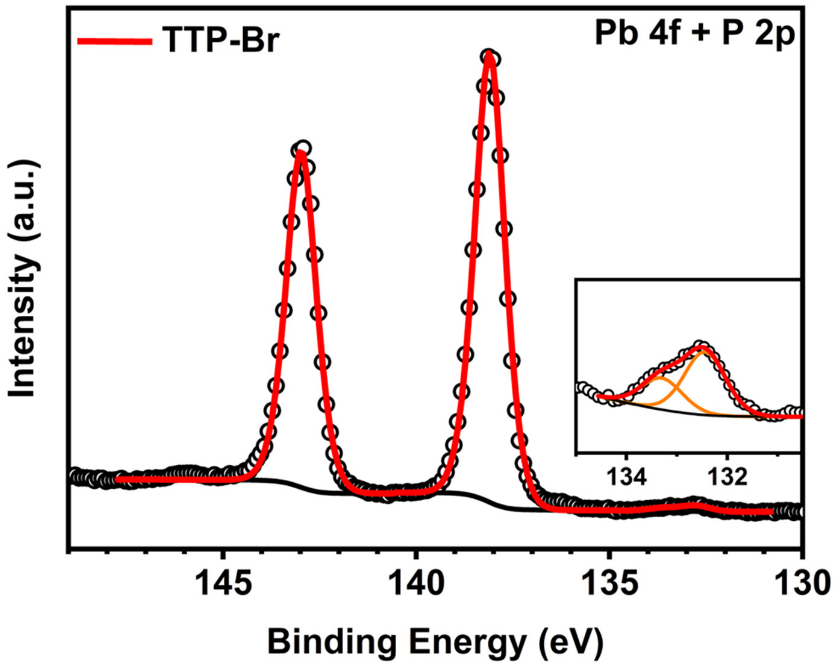

Figure S6. Pb 4*f* and P 2*p* (inset) XPS spectra of TTP-Br-capped $CsPbBr_3$ NCs.

Table S3. Elemental composition of the area shown in **Figure S7** performed via STEM-EDS.

Map Sum Spectrum	Line Type	weight%	atomic%	rel. error in % (1 Sigma)
**P**	K series	0.36	1.40	11.81
**Br**	K series	36.89	55.16	3.14
**Cs**	L series	22.53	20.25	10.15
**Pb**	L series	40.22	23.19	10.15
**Total**		100.00	100.00	

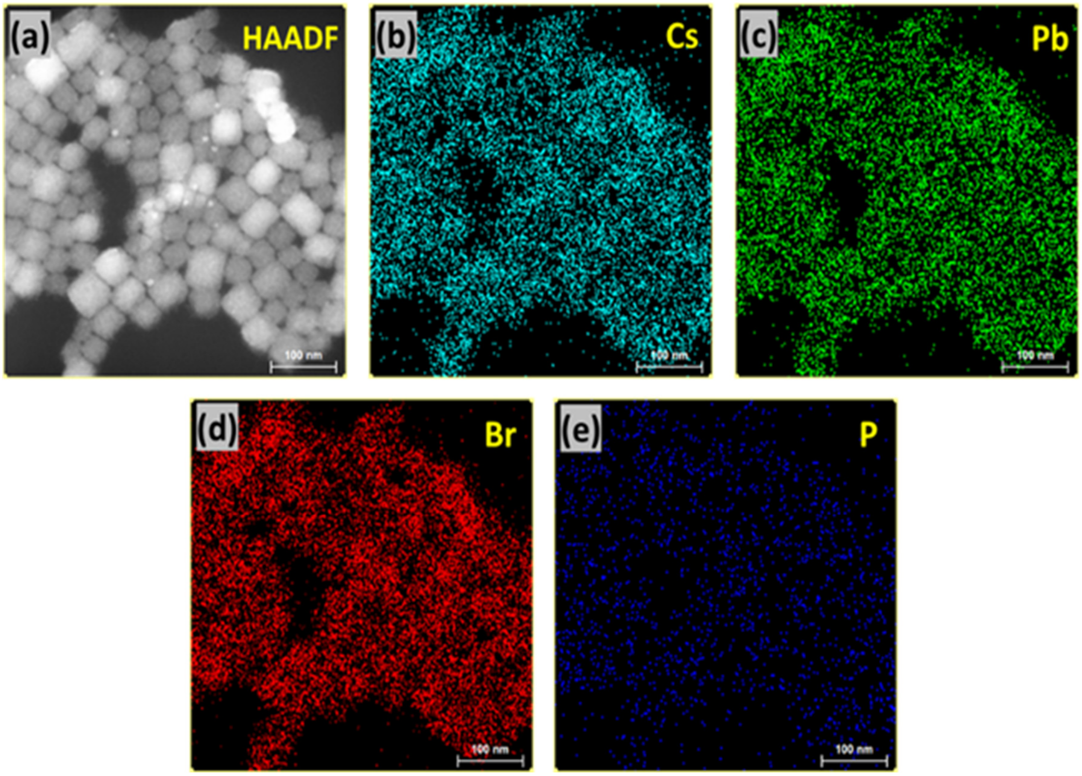

Figure S7. (a) Scanning transmission electron microscopy (STEM)-High-angle annular dark field (HAADF) image of TTP-Br-capped $CsPbBr_3$ NCs. (b-e) Corresponding elemental mappings of, Cs, Pb, Br, and P of TTP-Br-capped $CsPbBr_3$ NCs by energy-dispersive X-ray spectroscopy (EDS).

Table S4. Elemental analysis of the area shown in **Figure S8** derived from SEM-EDS.

**Map Sum Spectrum**	**Line Type**	**Wt%**	**Wt% Sigma**	**Atomic %**
**P**	K series	1.34	0.03	4.90
**Br**	K series	39.14	0.12	55.56
**Cs**	L series	22.73	0.08	19.40
**Pb**	L series	36.80	0.13	20.15
**Total**		100.00		100.00

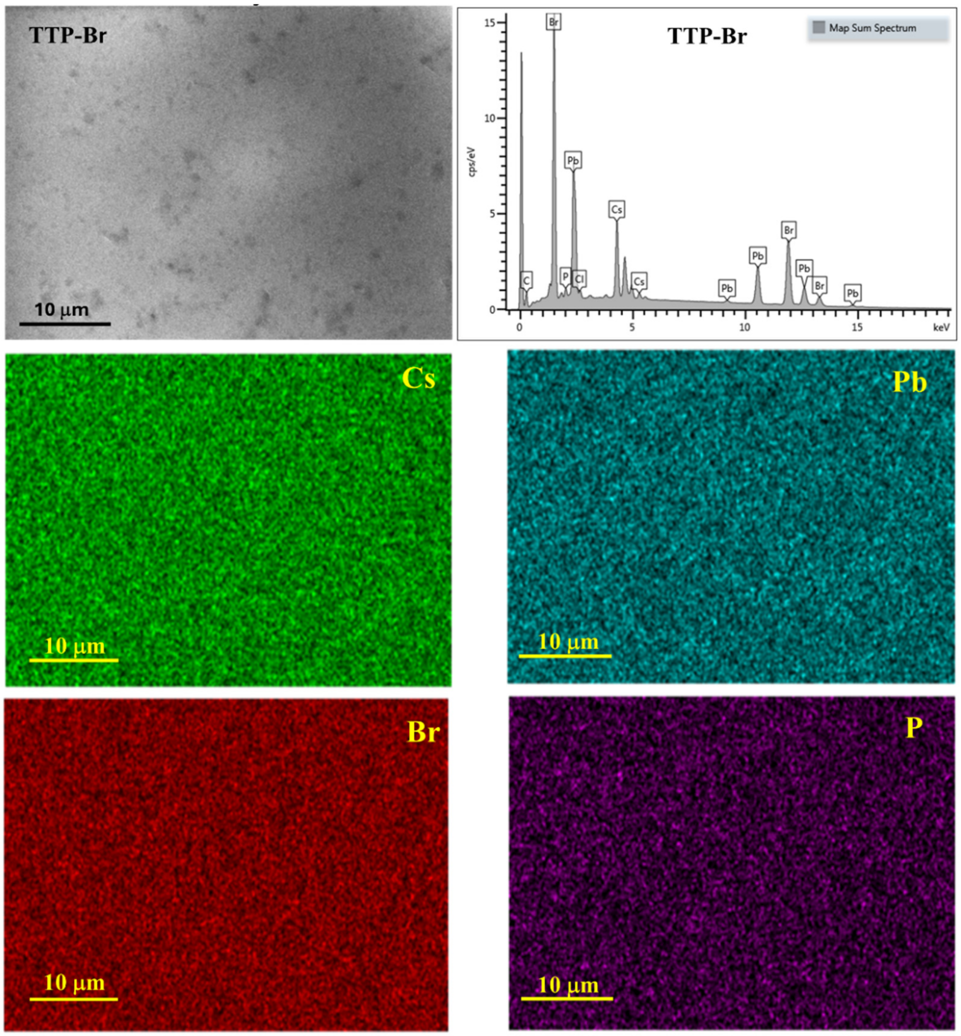

Figure S8. SEM-EDS elemental mapping of TTP-Br-capped $CsPbBr_3$ NCs.

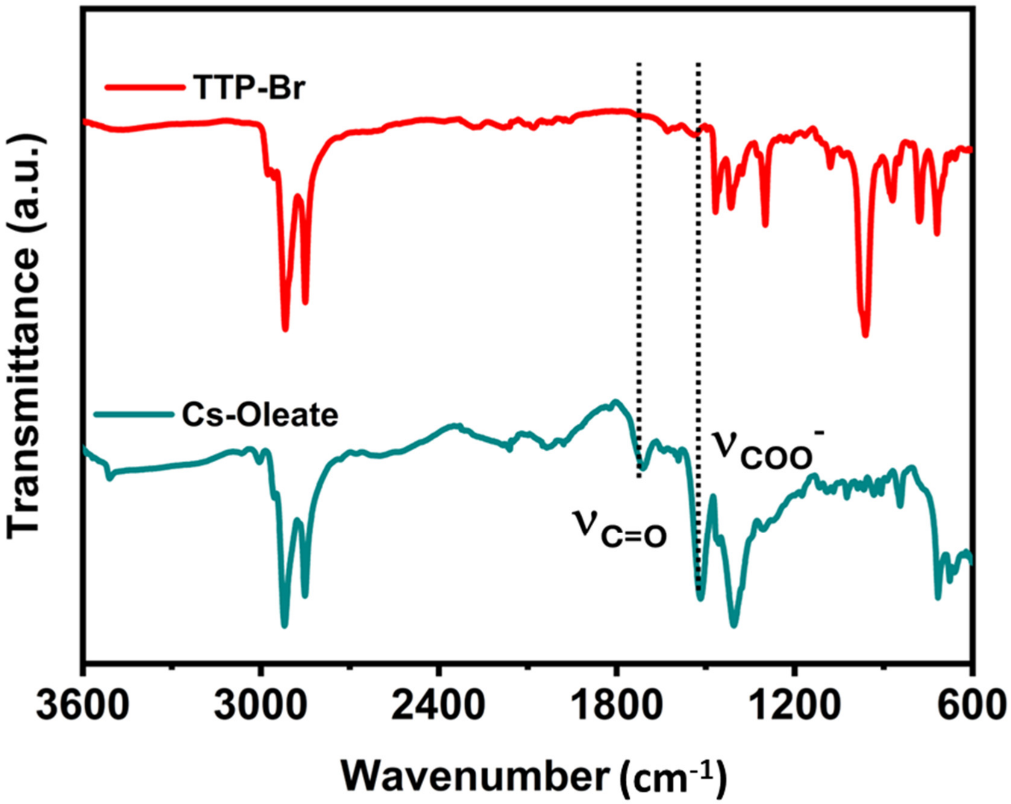

Figure S9. FT-IR spectra of TTP-Br-, and Cs-Oleate-capped $CsPbBr_3$ NCs.

.

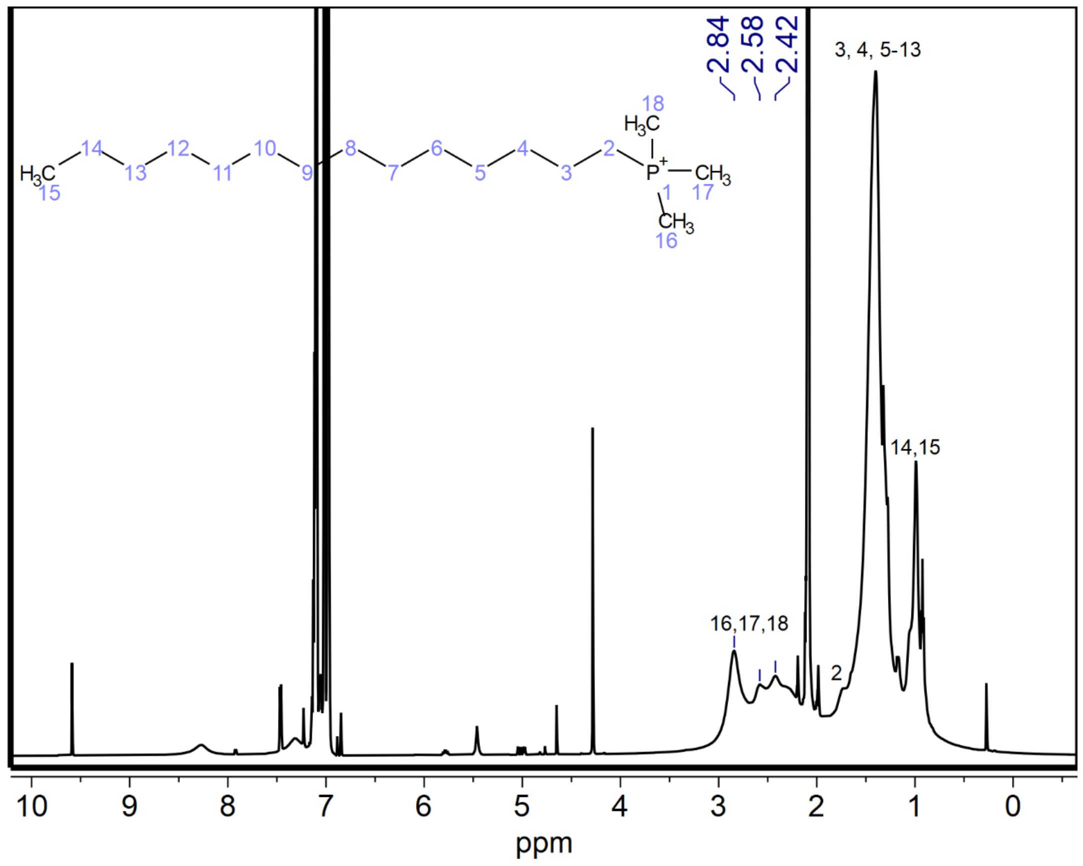

Figure S10. ^{1}H NMR spectrum of TTP-Br-capped $CsPbBr_3$ NCs in toluene-D at 298K, with diagnostic peak assignment, the TTP-Br structure formula, and signal numbering.

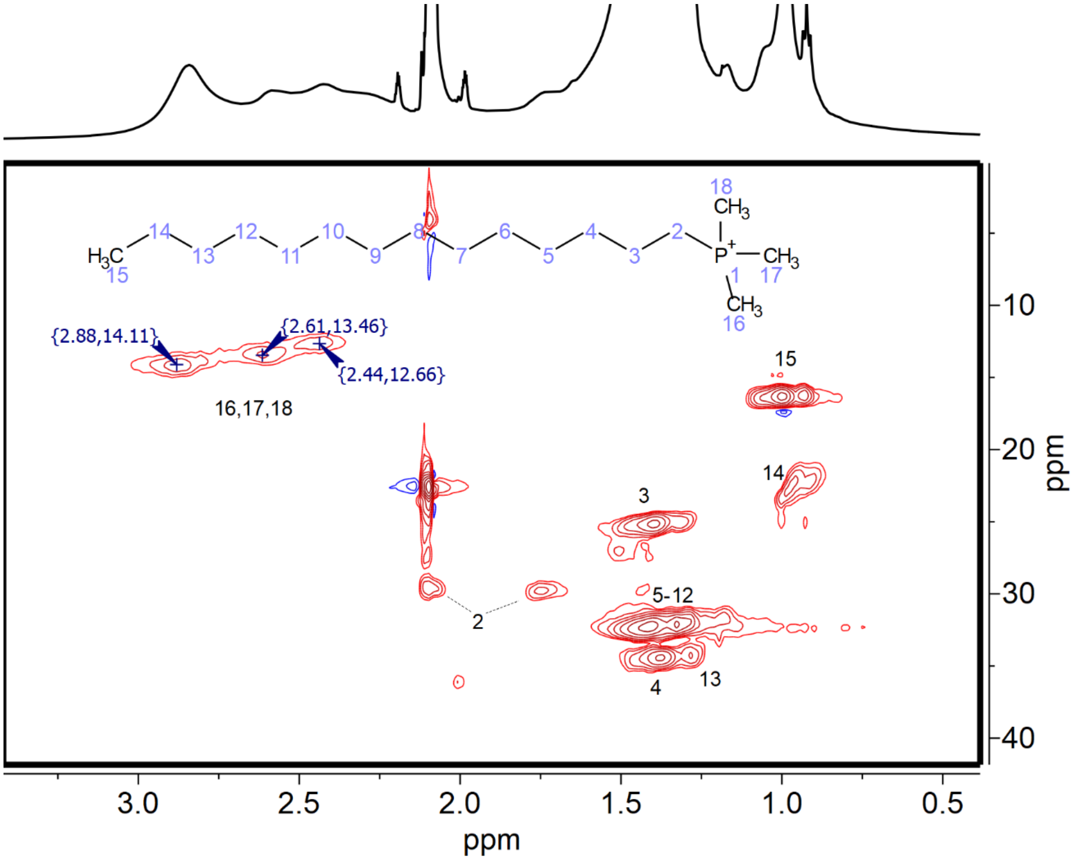

Figure S11. ^{1}H-^{13}C HSQC NMR spectrum of TTP-Br-capped $CsPbBr_3$ NCs in toluene-D at 298K, with peak assignment, the TTP-Br structure formula, and signal numbering.

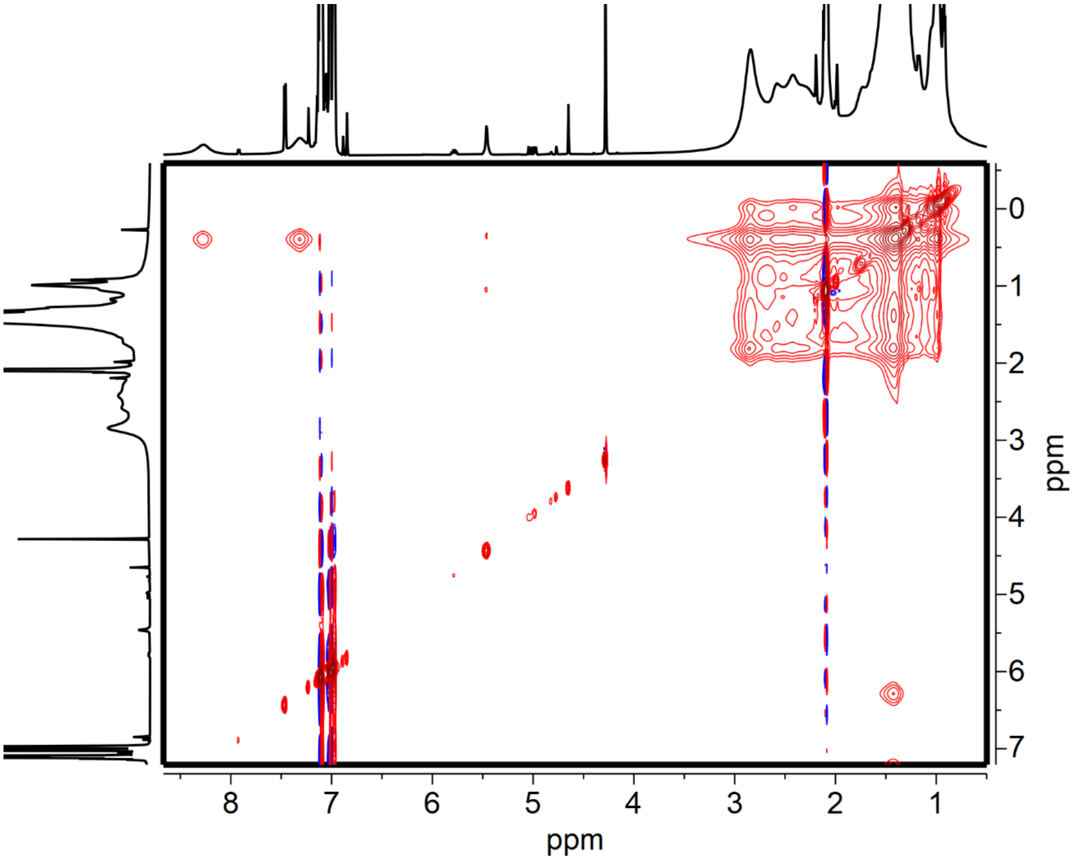

Figure S12. ^{1}H-^{1}H NOESY NMR spectrum of TTP-Br-capped $CsPbBr_3$ NCs in toluene-D at 298 K, negative (red) NOE cross peaks are typical of species with slow tumbling regime in solution, due to the binding with the NC's surface.

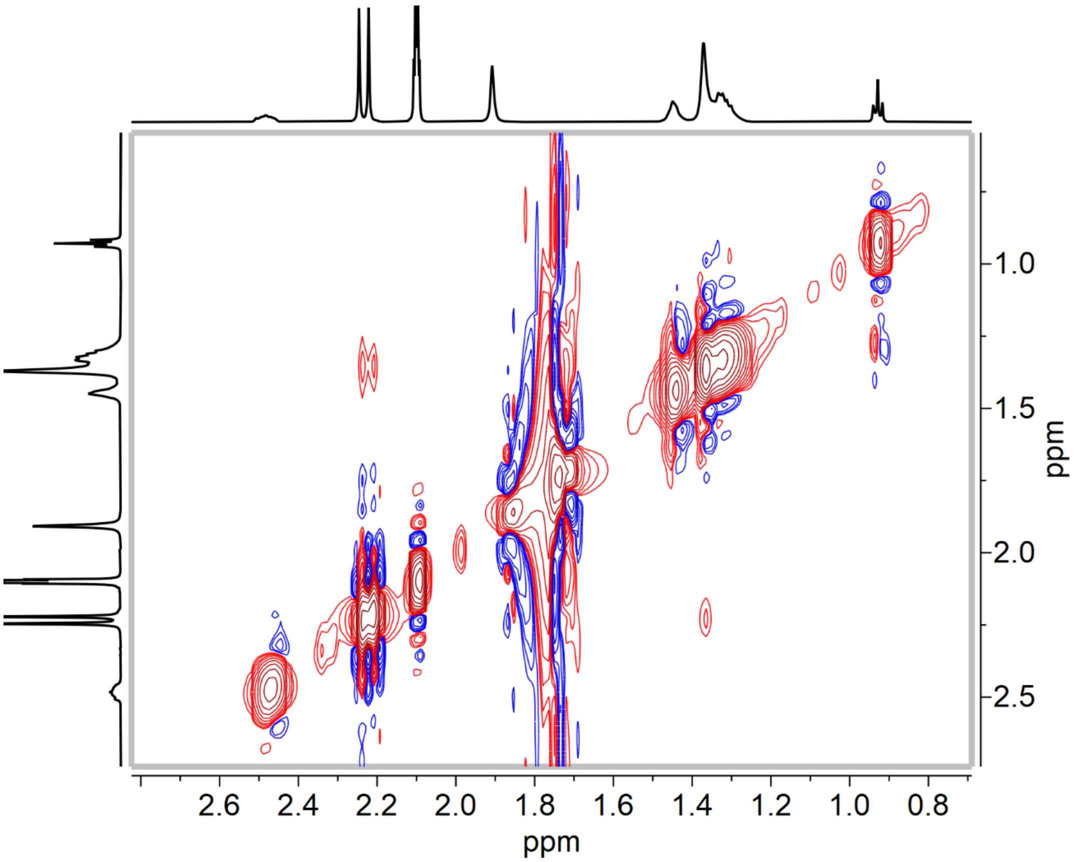

Figure S13. ^1H-^1H NOESY NMR spectrum of TTP-Br in toluene-D at 298 K, which shows negative (red) NOE cross peaks, likely due to micelles or aggregates formation. The same experiment at 313 K returns positive (blue) NOE cross peaks (**Figure S15**).

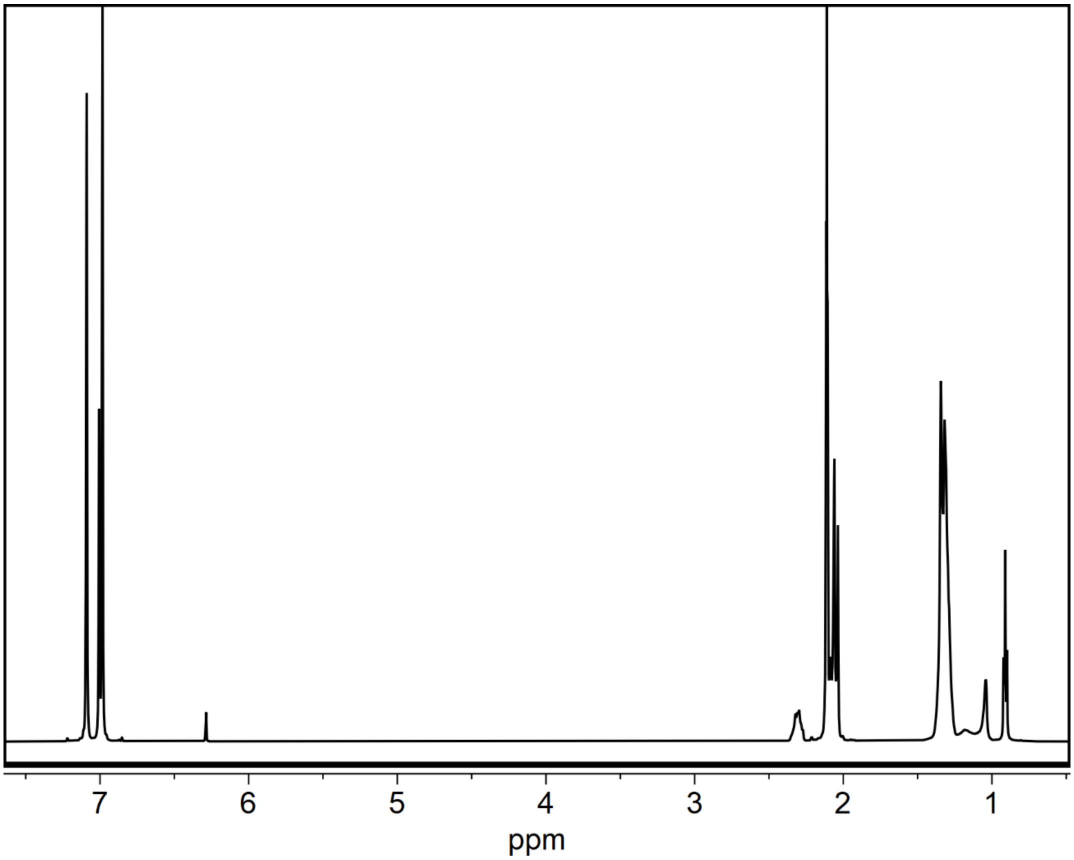

Figure S14. ^{1}H NMR spectrum of TTP-Br in toluene-D at 313 K.

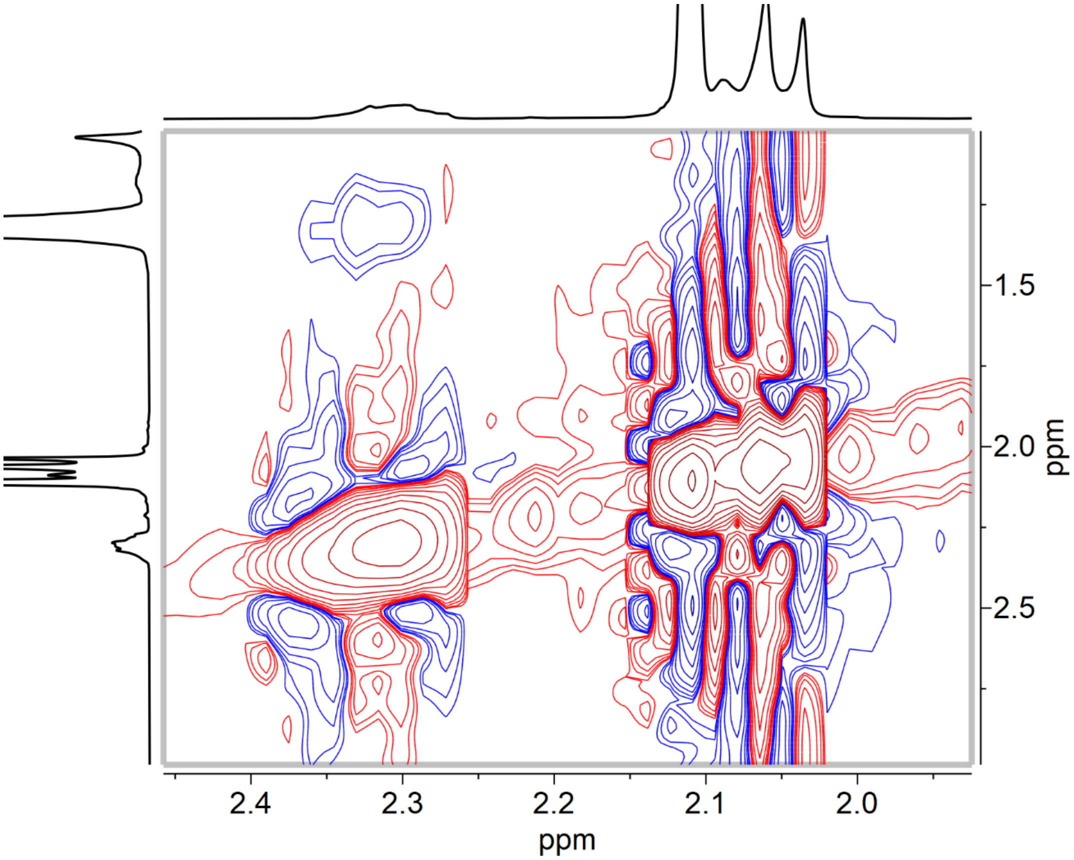

Figure S15. ^{1}H-^{1}H NOESY NMR spectrum of TTP-Br in toluene-D at 313K, which shows positive (blue) NOE cross peaks, typical of species with the fast-tumbling regime in solution.

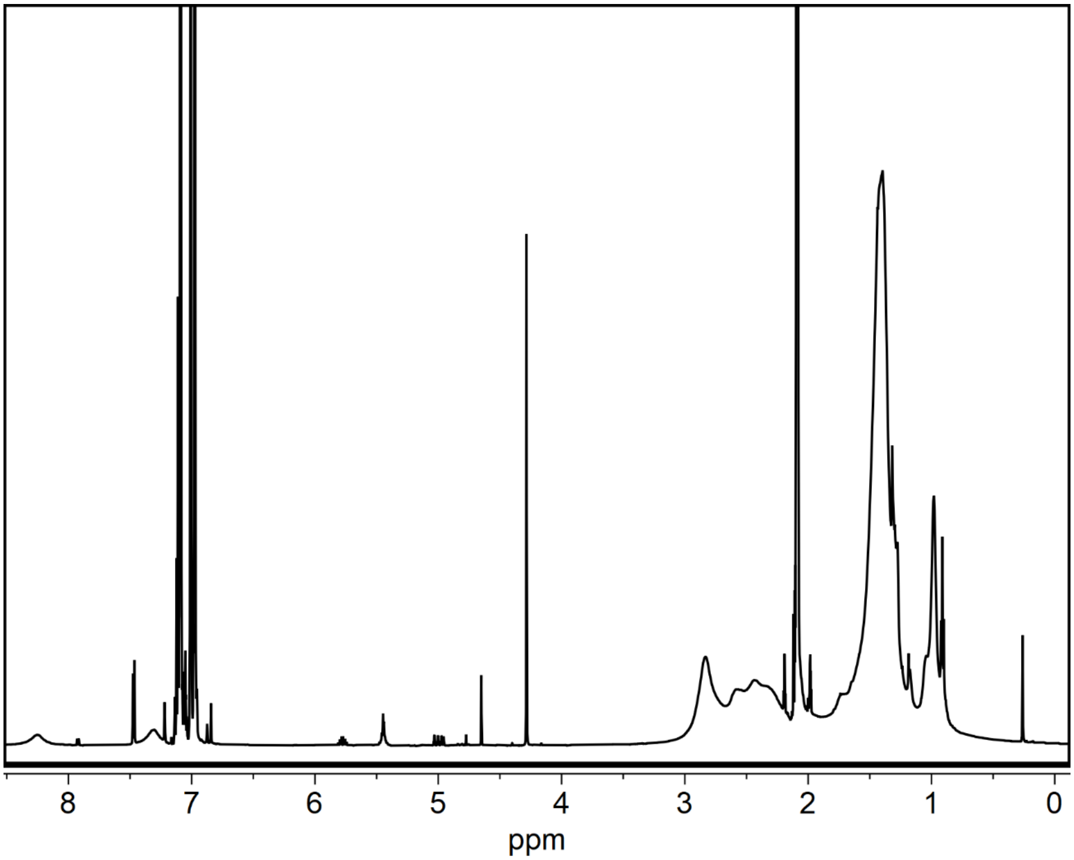

Figure S16. ^{1}H NMR spectrum of TTP-Br-capped $CsPbBr_3$ NCs in toluene-D at 313K.

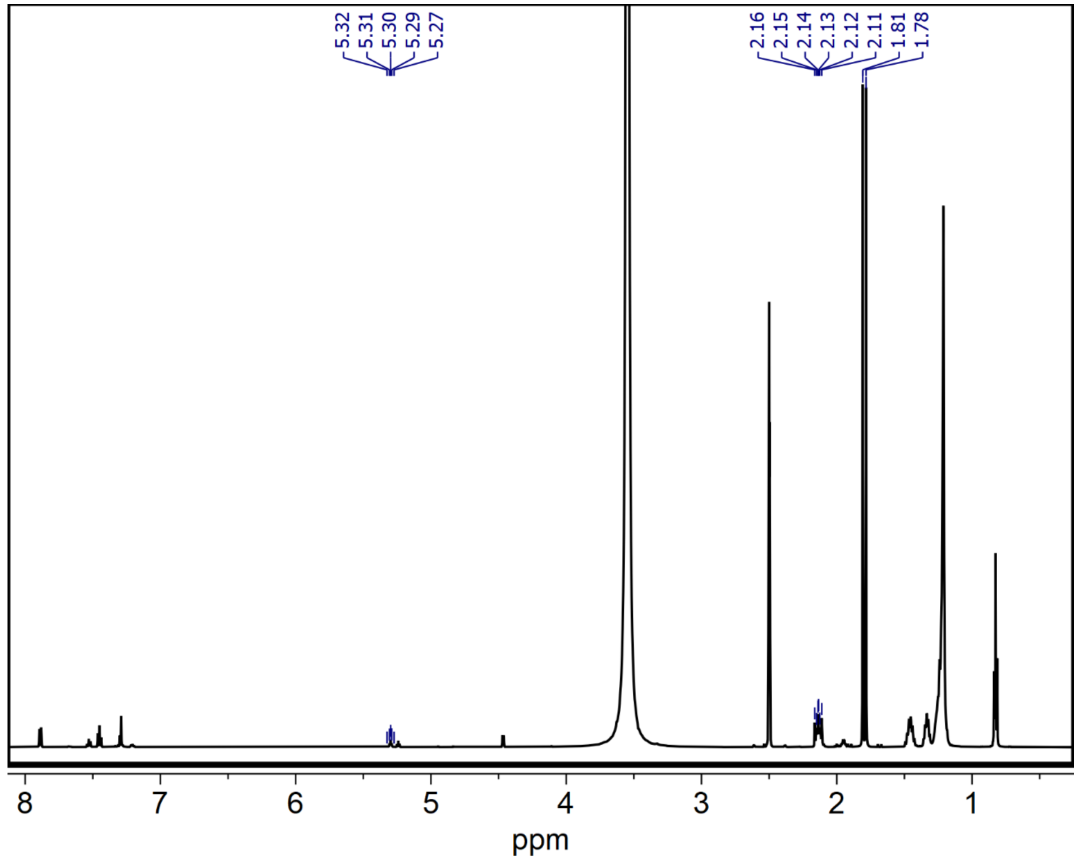

Figure S17. *quantitative* ^{1}H NMR spectrum of TTP-Br ligand in DMSO-*d6*, at 298K, ligand concentration is determined by comparing diagnostic integrated ligand peaks to that of a 10 mM standard dimethyl sulfone solution by using the PULCON method.[7]

Table S5. Summary of the parameters employed for the ligand density calculation.

	TTP-Br-capped NCs		DDA-Br-capped NCs	
	**Oleate**	**TTP-Br**	**Oleate**	**DDABr**
**[Pb] - ICP-OES (M)**	1.22E-04	1.22E-04	3.19E-05	3.19E-05
**[Pb]-DMSO (M)**	2.44E-01	2.44E-01	6.38E-02	6.38E-02
**N^o Pb atoms**	7.36E+18	7.36E+18	1.92E+18	1.92E+18
**NCs size (nm)**	8.9	8.9	8.8	8.8
**Unit cell size (nm)**	5.87E-01	5.87E-01	5.87E-01	5.87E-01
**Unit cells/side**	1.52E+01	1.52E+01	1.50E+01	1.50E+01
**N^o Pb atoms/ NC**	3.49E+03	3.49E+03	3.37E+03	3.37E+03
**N^o NCs**	2.11E+15	2.11E+15	5.70E+14	5.70E+14
**Total NC surface (nm^2)**	1.00E+18	1.00E+18	2.65E+17	2.65E+17
**[Ligands] (M)**	8.65E-04	1.07E-02	5.90E-04	2.91E-03
**N^o ligand molecules**	1.04E+17	1.29E+18	7.11E+16	3.50E+17
**Ligands density (Ligands /nm^2)**	**0.10**	**1.28**	**0.27**	**1.32**
**Surface Ligand %**		**92.52**		**83.12**

The Pb and the free ligands concentration were measured by ICP-OES and NMR, respectively, on the samples dissolved in DMSO-d6, whereas the NC size was measured via TEM analysis. The unit cell size was obtained from reference.[13]

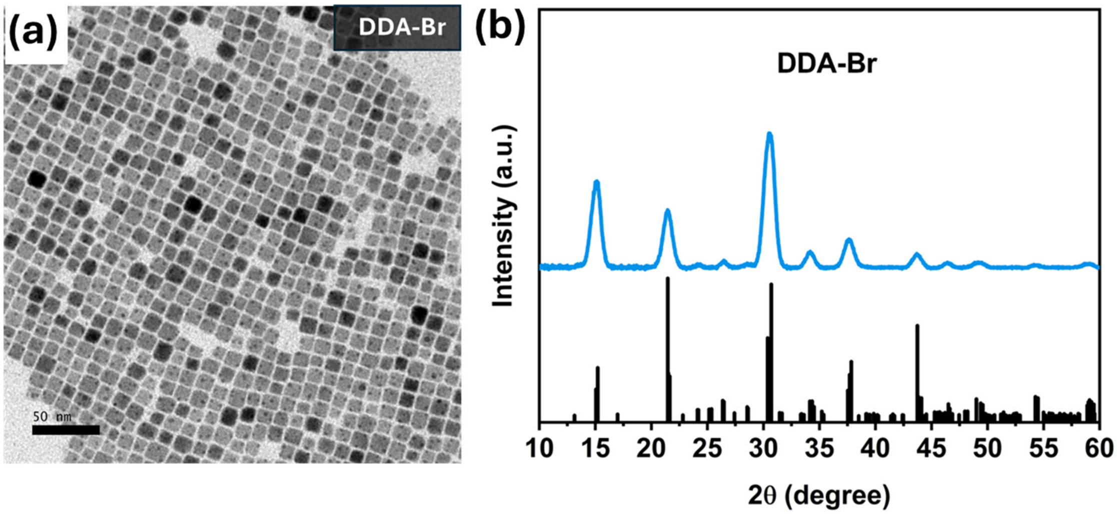

Figure S18. (a) TEM micrograph, and (b) XRD spectrum of DDA-Br-capped $CsPbBr_3$ NCs.

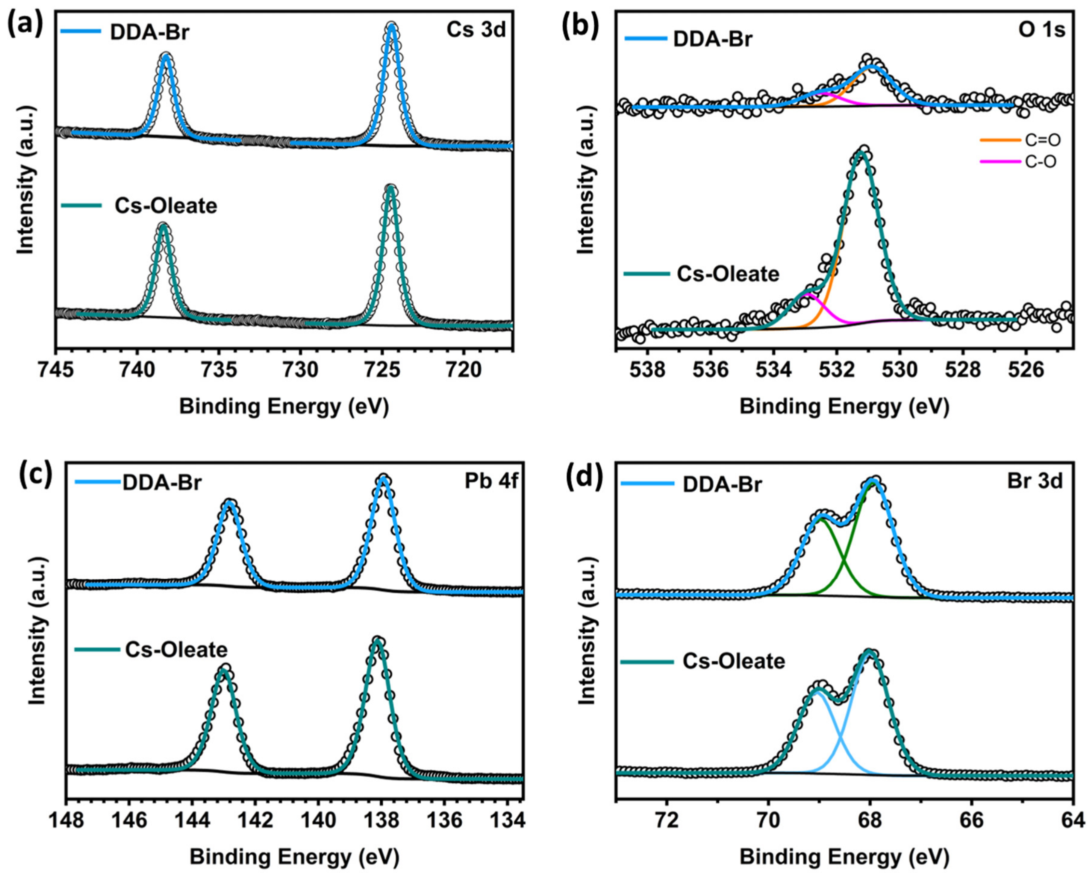

Figure S19. Comparison between (a) Cs 3*d*, (b) O 1*s*, (c) Pb 4*f*, (d) Br 3*d*, XPS spectra of DDA-Br-capped, and Cs-Oleate-capped $CsPbBr_3$ NCs.

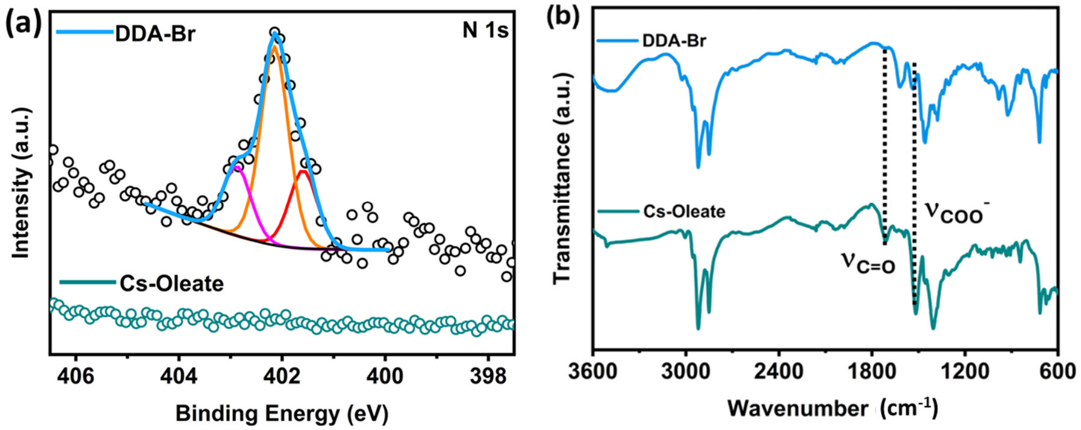

Figure S20. (a) Comparison between N 1*s* XPS spectra of DDA-Br- and Cs-Oleate-capped $CsPbBr_3$ NCs, (b) FTIR spectra, of DDA-Br- and Cs-Oleate-capped $CsPbBr_3$ NCs.

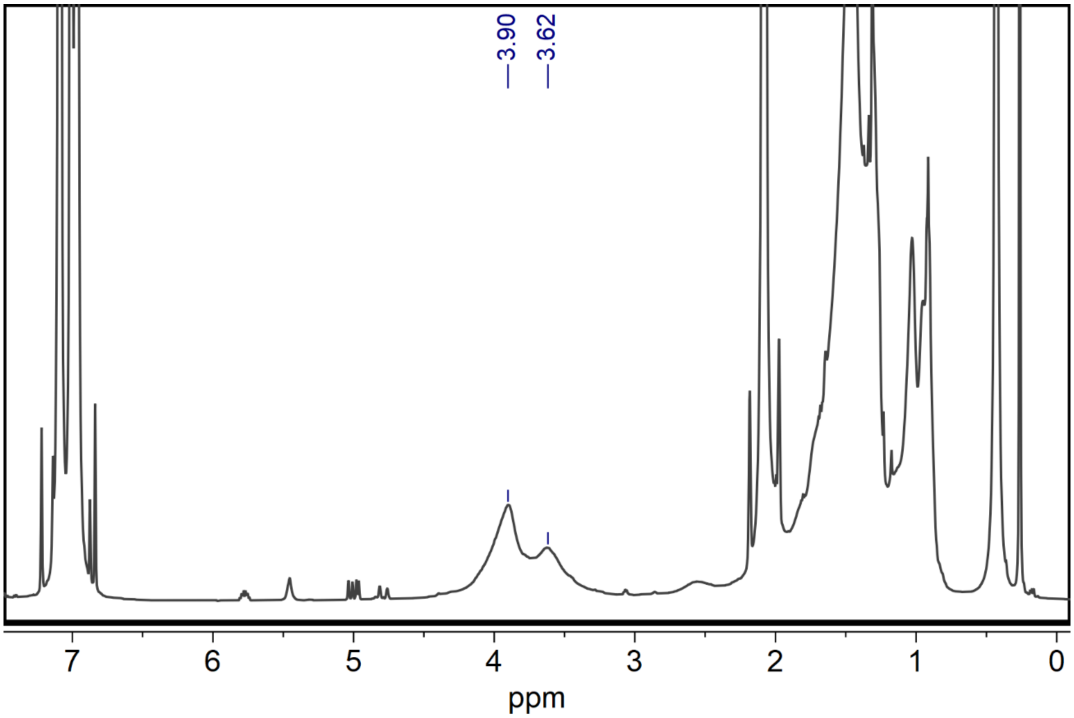

Figure S21. ^{1}H NMR spectrum of DDA-Br-capped $CsPbBr_3$ NCs in toluene-D at 298K.

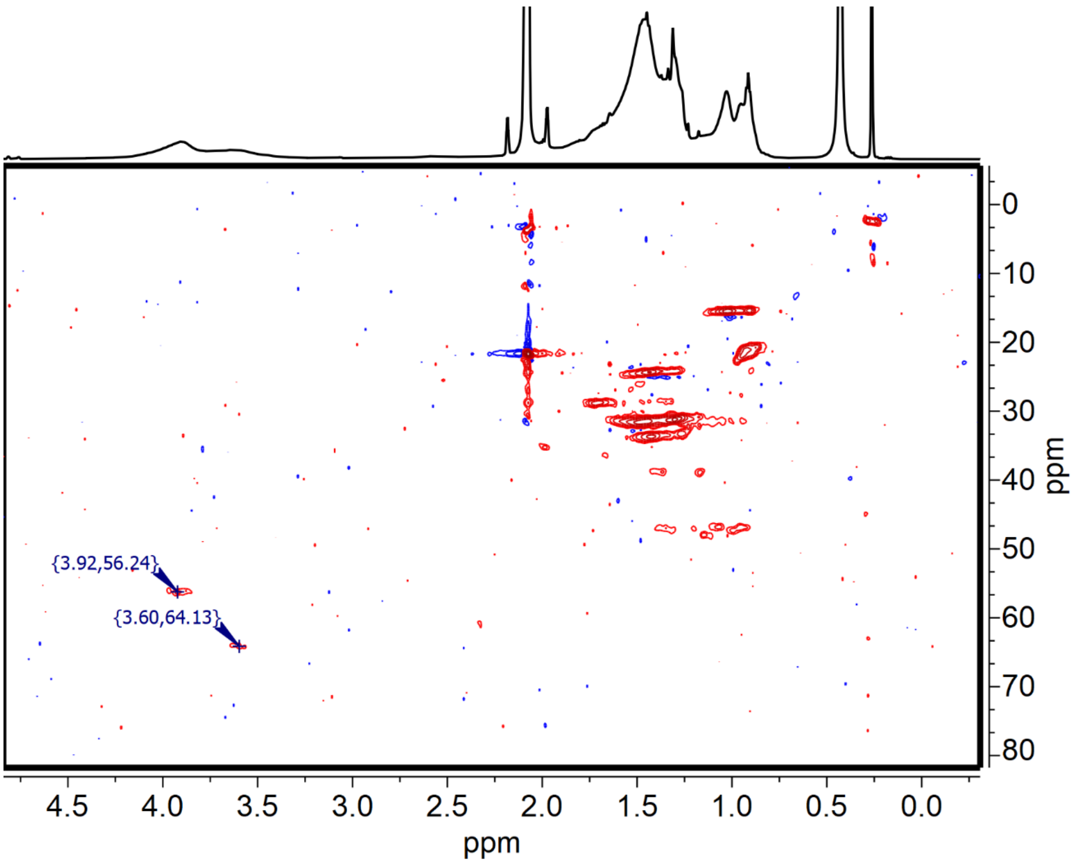

Figure S22. ^{1}H-^{13}C HSQC NMR spectrum of DDA-Br-capped $CsPbBr_3$ NCs in toluene-D at 298K.

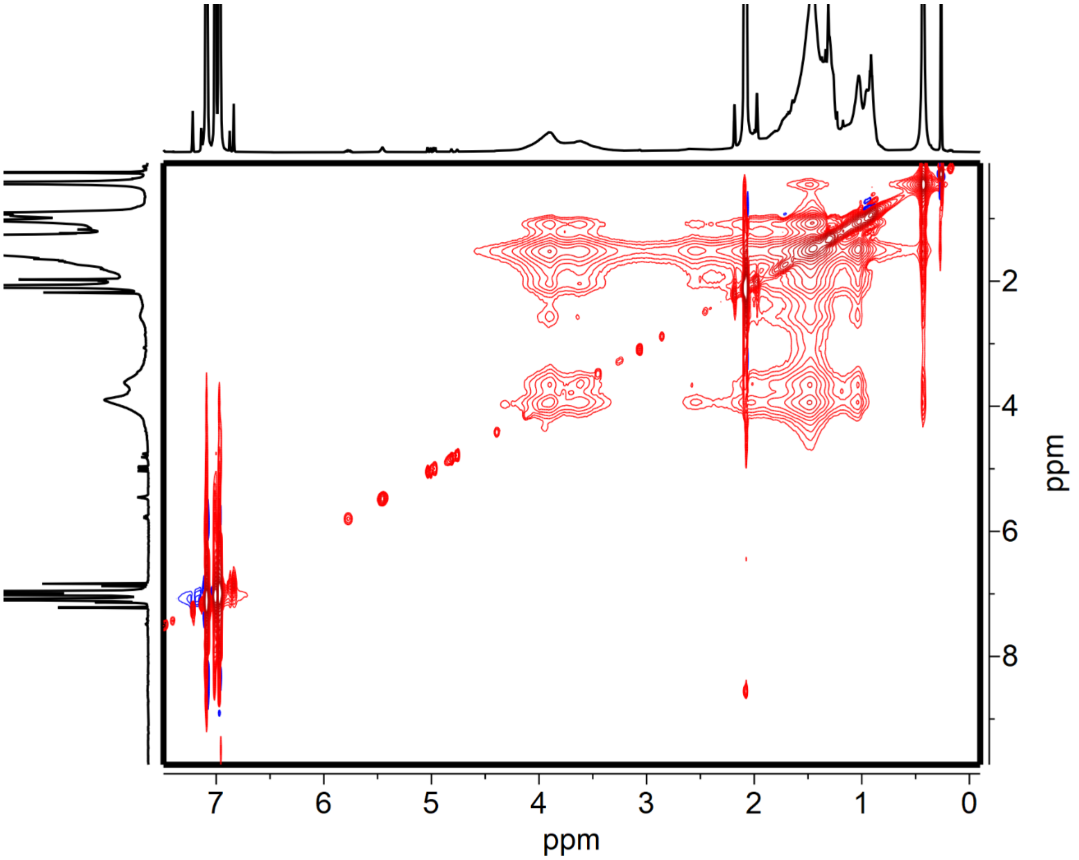

Figure S23. ^{1}H-^{1}H NOESY NMR spectrum of DDA-Br-capped $CsPbBr_3$ NCs in toluene-D at 298 K, negative (red) NOE cross peaks is typical of species with slow tumbling regime in solution, due to the binding with the NC's surface.

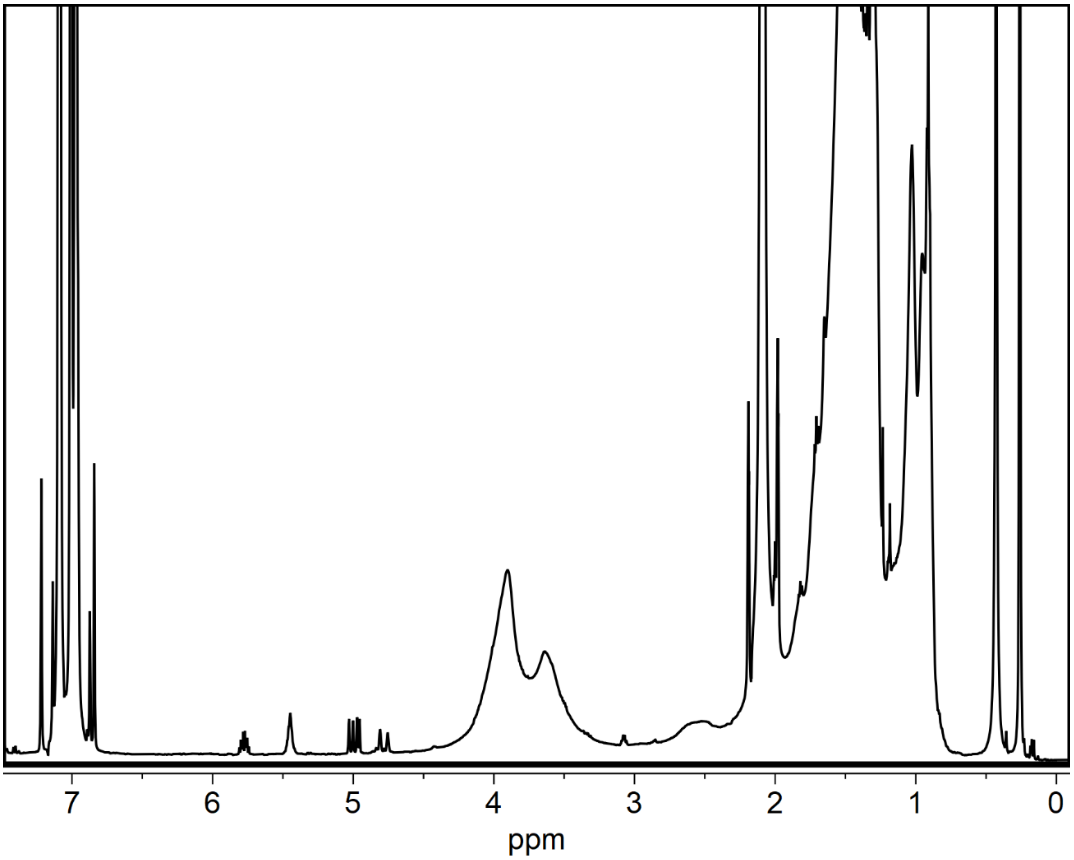

Figure S24. ^{1}H NMR spectrum of DDA-Br-capped $CsPbBr_3$ NCs in toluene-D at 313K.

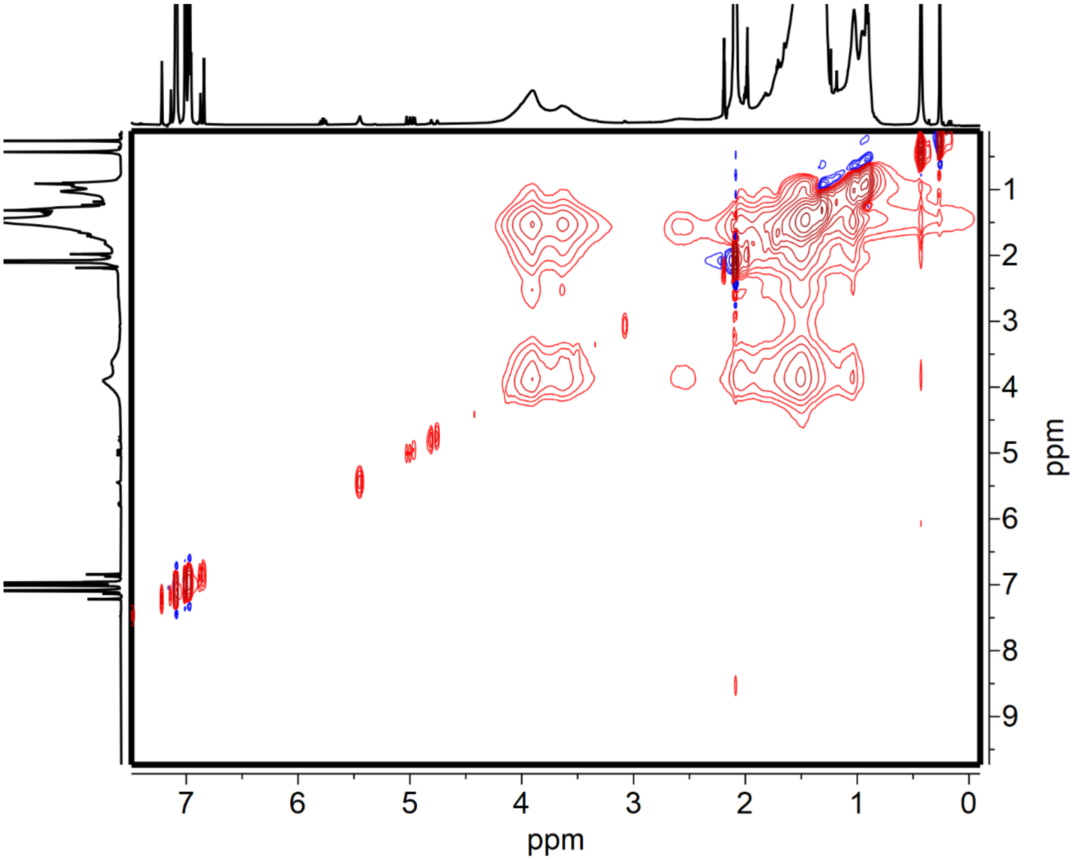

Figure S25. ^{1}H-^{1}H NOESY NMR spectrum of DDA-Br-capped $CsPbBr_3$ NCs in toluene-D at 313K, negative (red) NOE cross peaks is typical of species with slow tumbling regime in solution, due to the binding with the NC's surface.

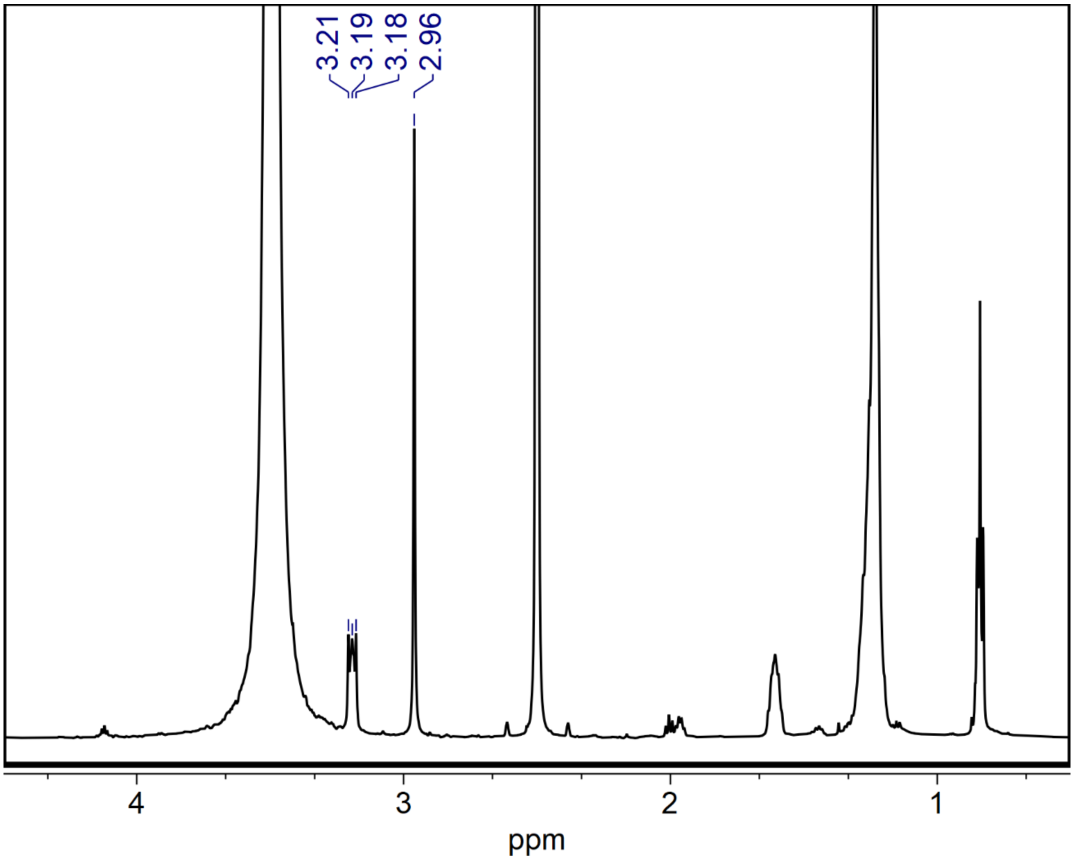

Figure S26. *quantitative* ^{1}H NMR spectrum of DDA-Br-capped $CsPbBr_3$ NCs in DMSO-*d6*, ligand concentration is determined by comparing diagnostic integrated ligand peaks to that of a 10 mM standard dimethyl sulfone solution, by using PULCON method.[7]

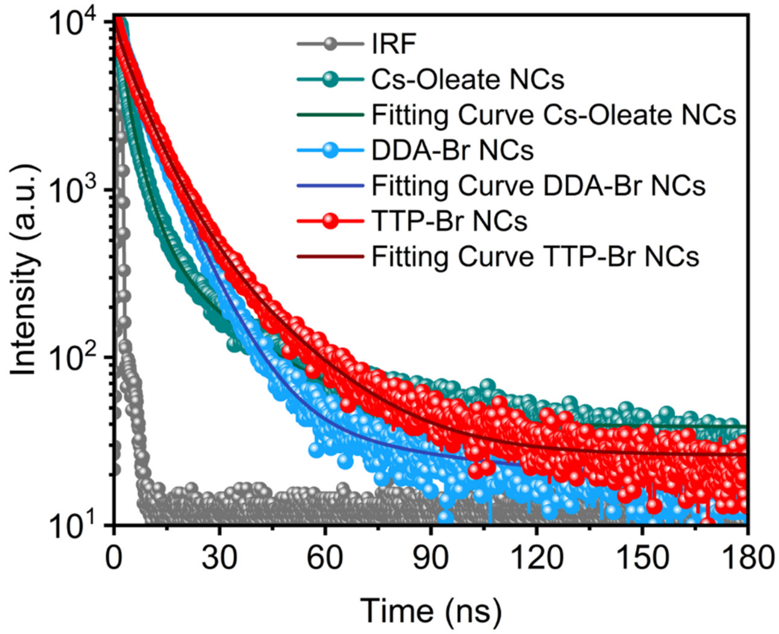

Figure 27: PL decay profile with fitting curves of Cs-Oleate-, DDA-Br-, and TTP-Br- capped $CsPbBr_3$ NCs.

Table: S6. TRPL fitting parameters and average PL lifetime (τ_{ave}) of the various $CsPbBr_3$ NCs samples.

**NCs Samples**	**A_1**	**τ_1 (ns)**	**A_2**	**τ_2 (ns)**	**A_3**	**τ_3 (ns)**	**R^2**	**τ_{ave} (ns)**
**Cs-Oleate**	5301	1.025	4350.8	4.13	570.01	20.05	0.99925	8.86
**DDA-Br**	5432.45	5.5991	4333.18	9.9490	38.56	75.83	0.99978	10.74
**TTP-Br**	1268.75	1.9233	7174.66	7.2464	1408.88	19.68	0.99971	11.29

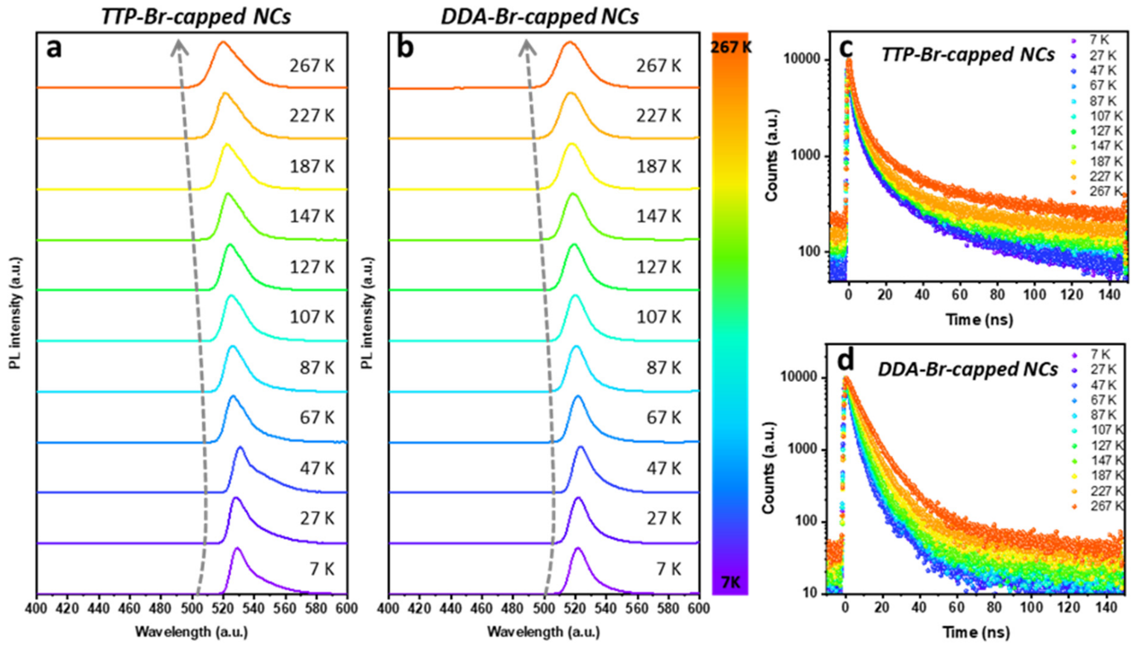

Figure S28. PL spectra of (a) TTP-Br-capped and (b) DDA-Br-capped $CsPbBr_3$ NC films recorded from 7 K to 267 K. PL decay traces of (c) TTP-Br-capped and (d) DDA-Br-capped $CsPbBr_3$ NC films recorded from 7 K to 267 K. For low temperature (7 K to 67 K), we are at the time resolution of our instrument.

Table: S7. TRPL fitting parameters and average PL lifetime (τ_{ave} in ns) of TTP-Br-capped $CsPbBr_3$ NCs thin film recorded at different temperatures.

**TTP-Br-capped $CsPbBr_3$ NCs Biexponential decay curve fitting**						
**Temperature (K)**	**A_1**	**τ_1 (ns)**	**A_2**	**τ_2 (ns)**	**R^2**	**$\tau_{Average}$ (ns)**
7	8095.12	1.31	2470.02	11.76	0.99562	8.97
27	7930.40	1.34	2354.04	11.67	0.99592	8.79
47	7868.33	1.40	2412.78	12.34	0.99562	9.39
67	7555.12	1.61	2624.13	12.54	0.99638	9.59
87	7736.04	1.64	2725.74	12.16	0.99658	9.25
107	7545.79	1.77	2751.27	12.33	0.99696	9.34
127	7697.44	1.73	2858.17	11.33	0.99685	8.53
147	7618.06	1.91	2798.72	11.86	0.99753	8.83
187	7620.62	2.09	2716.30	12.30	0.99757	9.01
227	7706.80	2.17	2656.23	12.94	0.99758	9.41
267	7682.56	2.56	2470.87	17.22	0.99736	12.58

Table: S8. TRPL fitting parameters and average PL lifetime (τ_{ave} in ns) of DDA-Br-capped $CsPbBr_3$ NCs thin film recorded at different temperatures.

**DDA-Br-capped $CsPbBr_3$ NCs Biexponential decay curve fitting**						
**Temperature (K)**	**A_1**	**τ_1 (ns)**	**A_2**	**τ_2 (ns)**	**R^2**	**$\tau_{Average}$ (ns)**
7	9877.25	2.91	1165.97	11.05	0.99969	5.43
27	9793.64	2.80	1286.31	10.37	0.99965	5.28
47	9887.97	2.71	1161.89	10.01	0.99975	4.92
67	9672.57	2.92	1455.09	10.34	0.9996	5.50
87	9679.35	3.25	1318.01	11.31	0.99942	5.84
107	9554.99	3.55	1335.15	11.44	0.99963	6.00
127	9832.43	3.88	1200.08	12.13	0.99942	6.16
147	9952.69	4.28	1007.83	13.54	0.99952	6.53
187	10368.92	5.42	546.58	19.79	0.99934	7.74
227	10302.02	6.09	617.28	20.99	0.99938	8.64
267	9947.19	7.30	878.38	22.30	0.99939	10.49

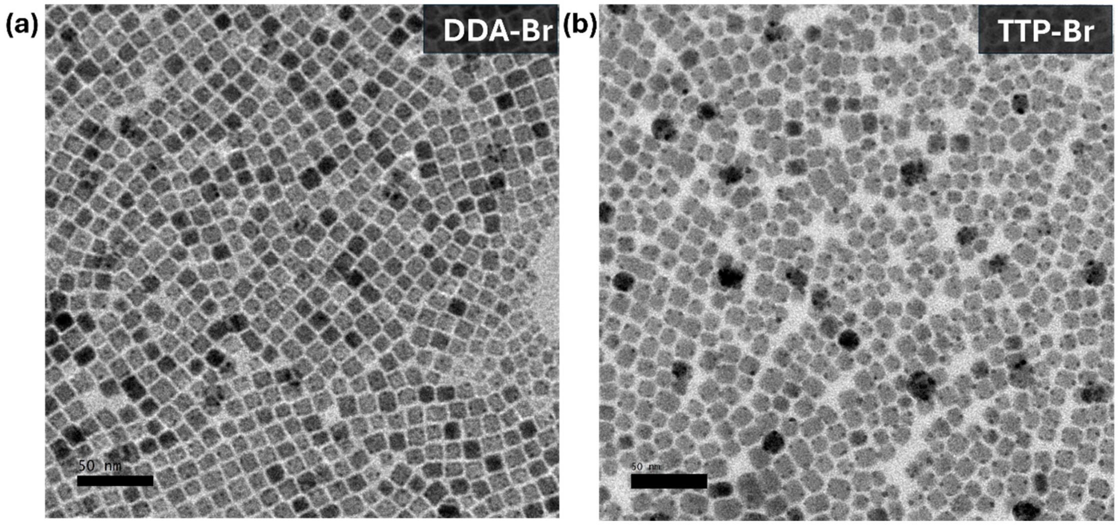

Figure S29. TEM micrographs of (a) DDA-Br-capped $CsPbBr_3$ NCs (average size of 10.3 ± 1.6 nm) and (b) TTP-Br-capped $CsPbBr_3$ NCs (average size of 9.8 ± 1.8 nm) after six weeks of storage under air.

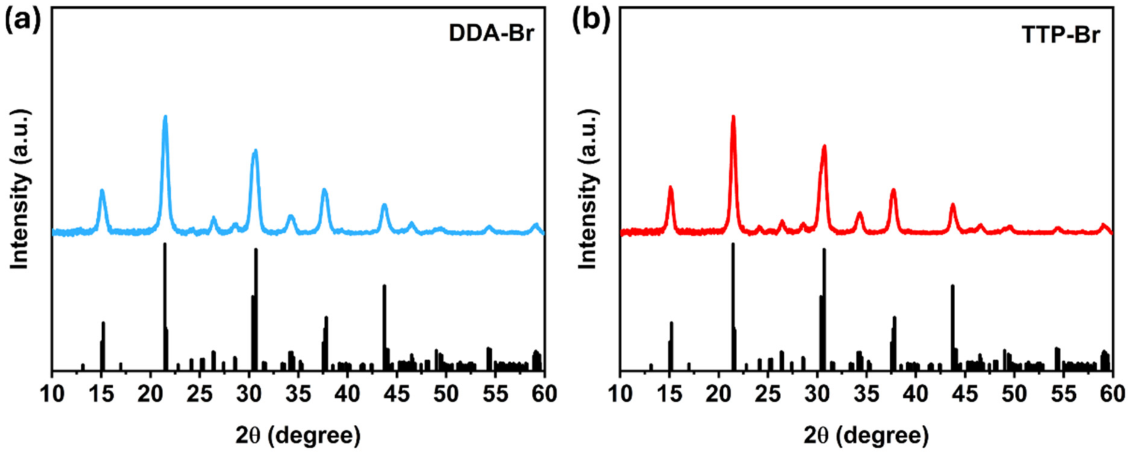

Figure S30. XRD spectra of (a) DDA-Br-capped, (b) TTP-Br-capped $CsPbBr_3$ NCs after six weeks of storage under air.

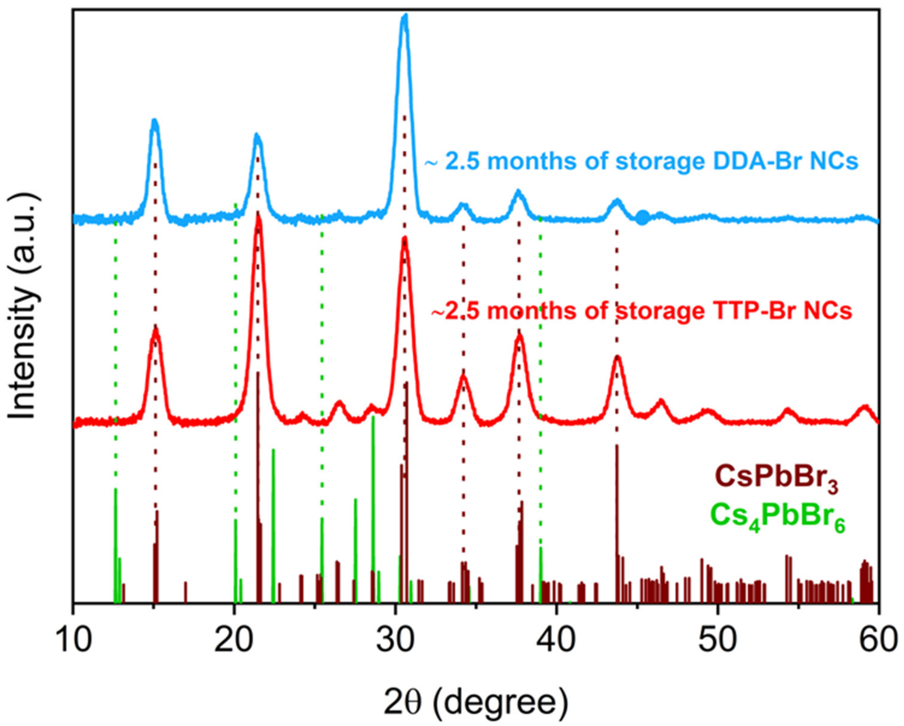

Figure S31: XRD spectra of TTP-Br-capped and DDA-Br-capped $CsPbBr_3$ NCs after storage of ~2.5 months at ambient air conditions.

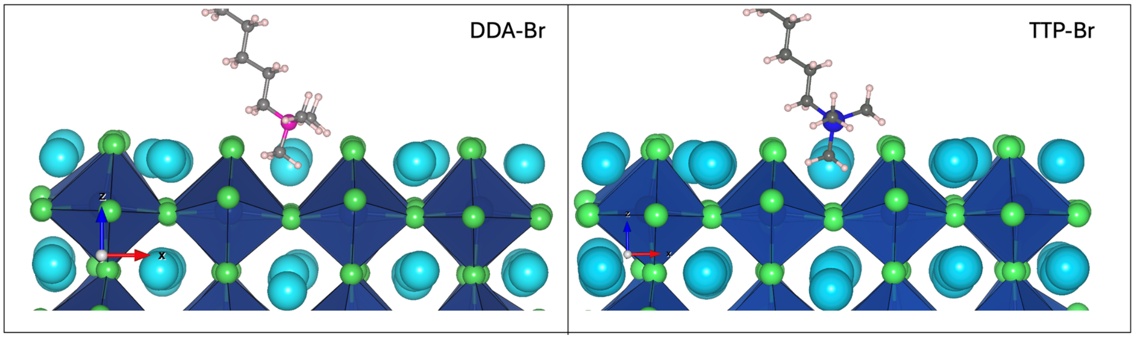

Figure S32. Binding configuration of (left) a DDA⁺ and (right) a TTP⁺ ligands sitting in the A-site of the $CsPbBr_3$ NCs' surface after structural relaxation at the DFT/PBE level. The quaternary ammonium and phosphonium ligands assume similar orientations, with one of the N-CH_3 or P-CH_3 bonds almost perpendicular to the NC surface.

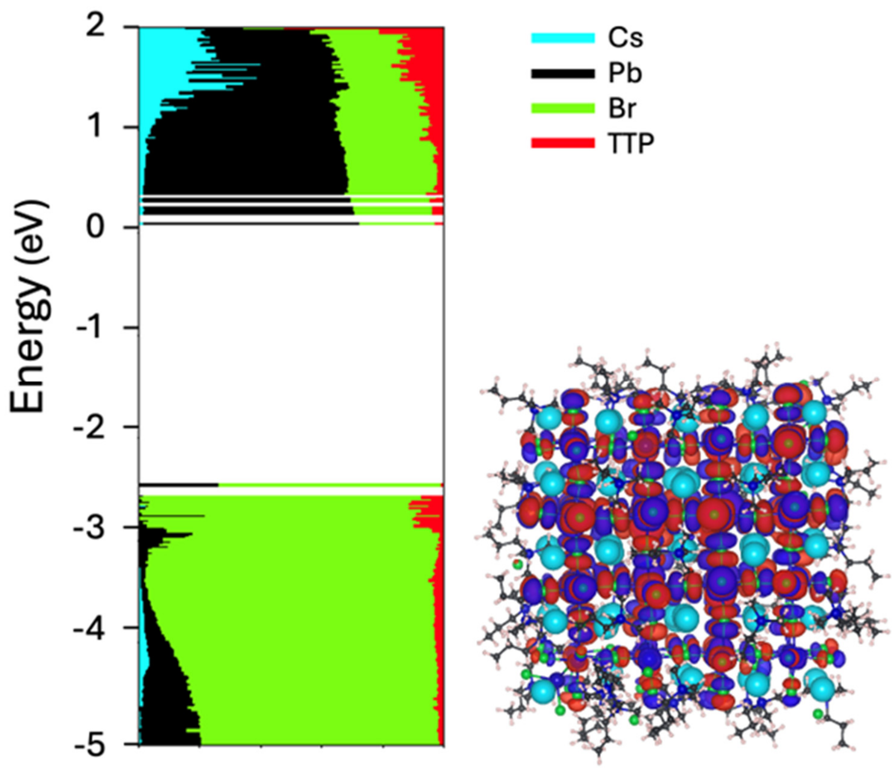

Figure S33. (left) Electronic structure of the ~2.4 nm-sided $CsPbBr_3$ NC model passivated with TTP-Br ligand with a surface concentration of 1.27 ligands/nm^2 computed at the DFT/PBE level of theory. The color code indicates the contribution of each atom type to each molecular orbital and the C, H and P contributions from TTP-Br ligands are grouped for clarity. (right) Isosurface of the valence band edge orbital with a counter value of 0.02 e/$Bohr^3$.

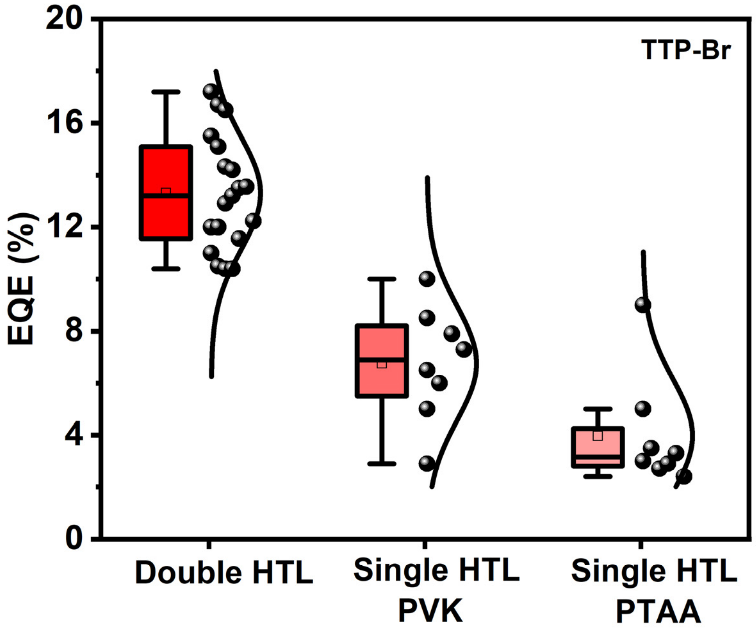

Figure S34. Statistics of EQE values of TTP-Br-capped $CsPbBr_3$ NCs-based LEDs having either a double or single HTL configuration.

Table S9. UPS analysis of $CsPbBr_3$ NCs.

$CsPbBr_3$ NCs	WF	E_{ion}	$E_1 = E_{ion}$ - WF
**TTP-Br**	4.52	5.31	0.79
**DDA-Br**	4.57	5.30	0.73

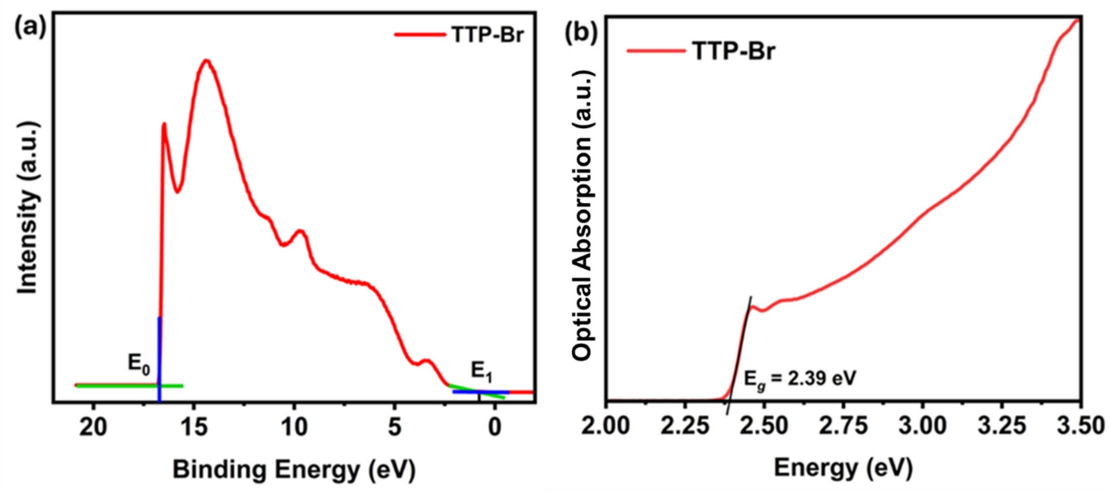

Figure S35. (a) UPS spectrum, (b) absorption spectrum of TTP-Br-capped $CsPbBr_3$ NCs.

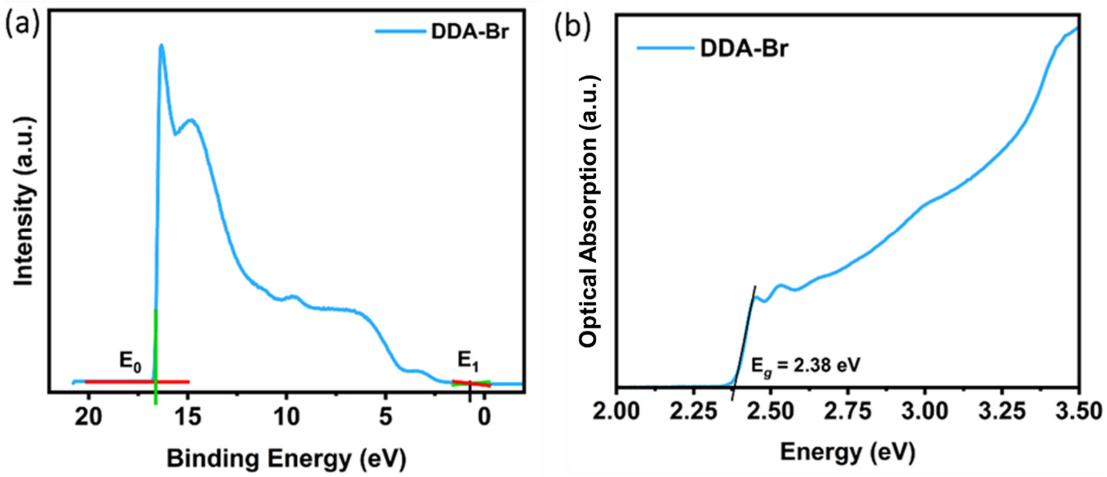

Figure S36. (a) UPS spectrum, (b) absorption spectrum of DDA-Br-capped $CsPbBr_3$ NCs.

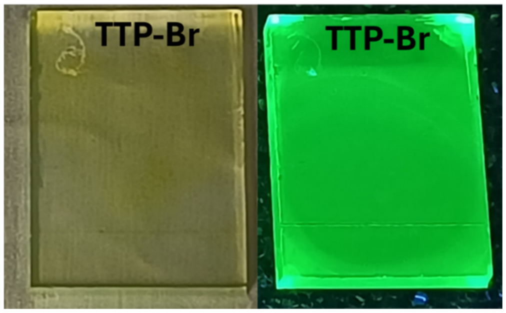

Figure S37. TTP-Br-capped $CsPbBr_3$ NC films under normal light and under UV light.

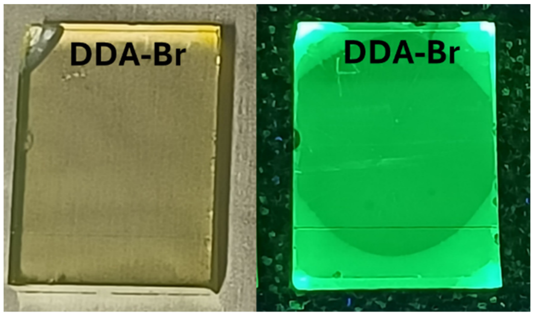

Figure S38. DDA-Br-capped $CsPbBr_3$ NC films under normal light and under UV light.

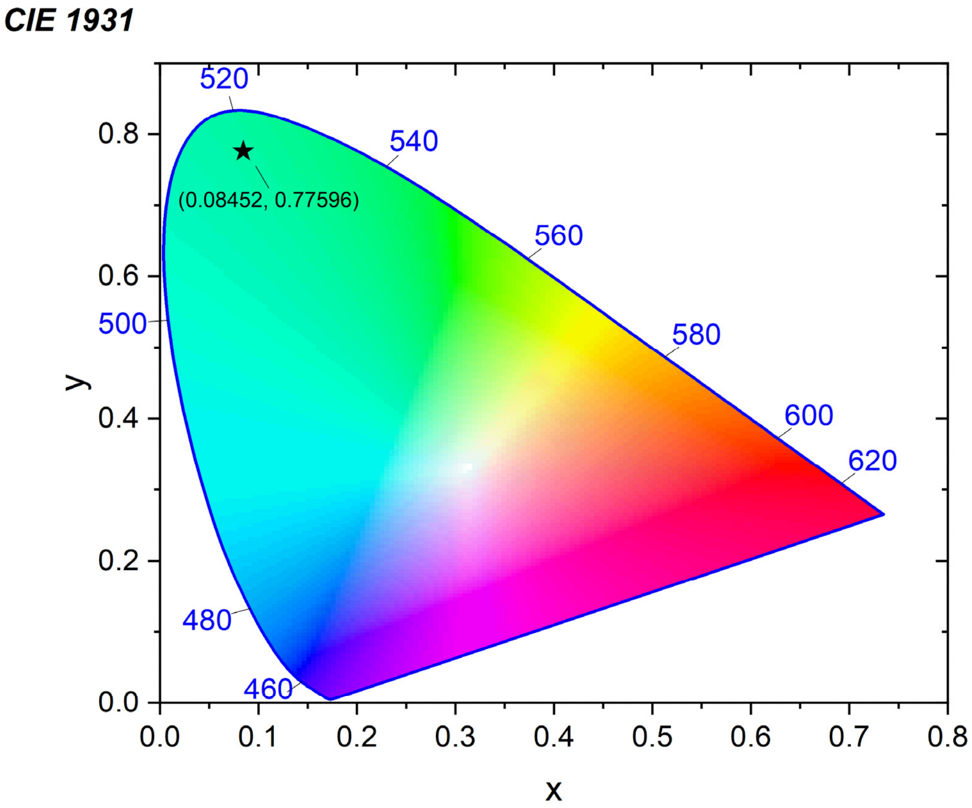

Figure S39. Commission Internationale de 1'Eclairage (CIE) 1931 colour coordinates.

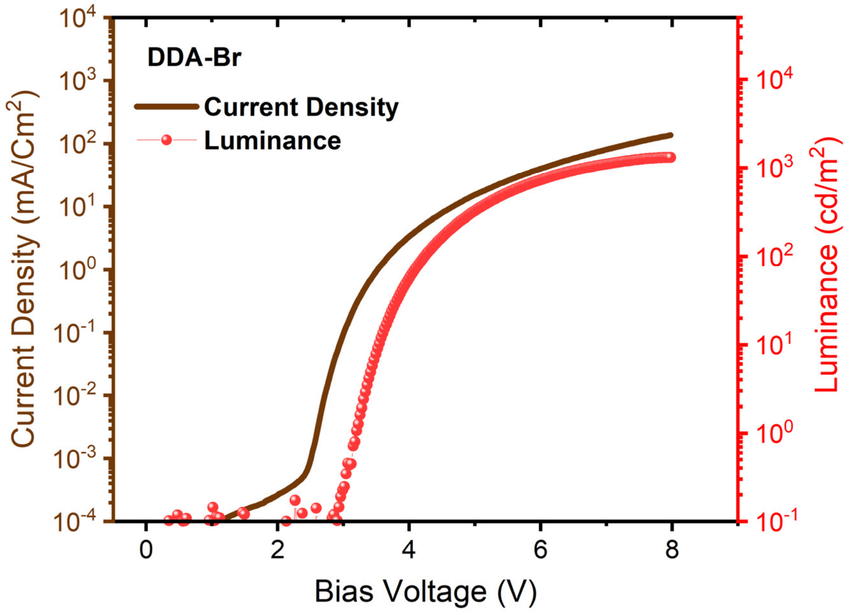

Figure S40. Current density and luminescence versus driving voltage curves of the DDA-Br-capped $CsPbBr_3$ NCs LED with double HTL.

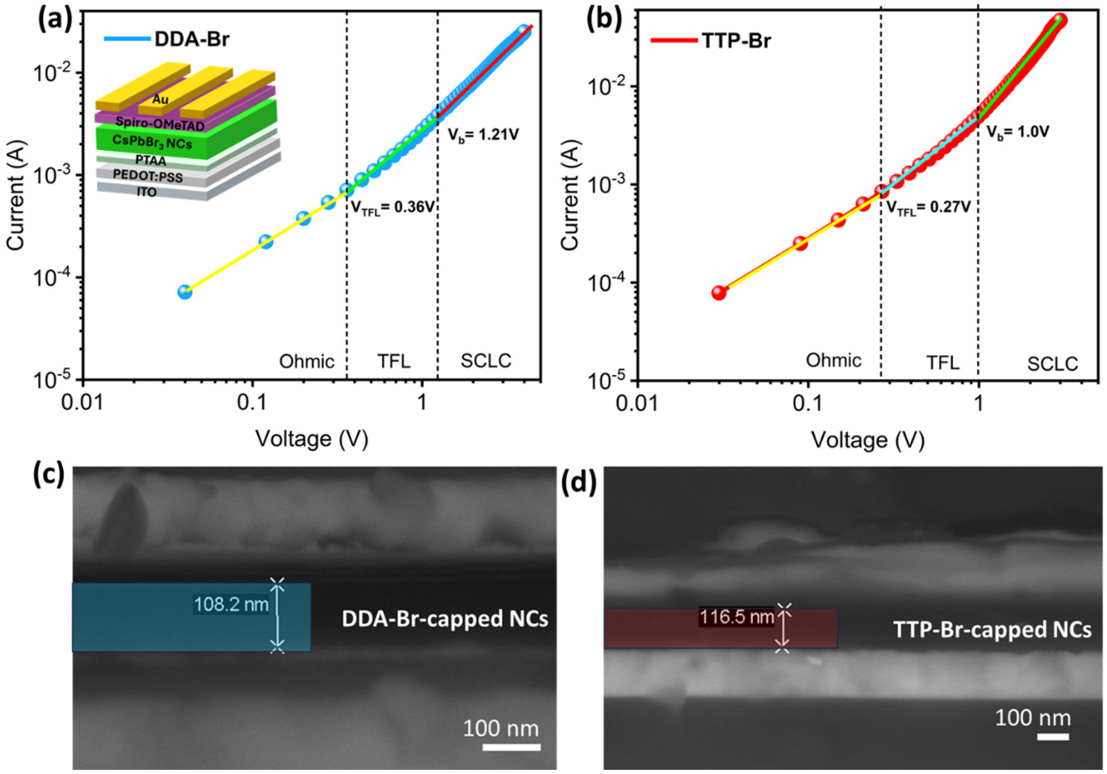

Figure S41. Space charge limit current (SCLC) measurements of the hole-only devices, with the structure (a) DDA-Br-capped, (b) TTP-Br-capped $CsPbBr_3$ NCs. Cross-sectional SEM images of the thin films with (c) DDA-Br-capped, and (d) TTP-Br-capped $CsPbBr_3$ NCs.

The hole mobility was determined by the Mott-Gurney equation[14]:

$$J_D = \frac{9\varepsilon\varepsilon_0\mu {V_b}^2}{8L^3}$$

where ε is the relative permittivity of the $CsPbBr_3$ NCs (4.8),[15] ε_0 is the vacuum permittivity (8.85 x 10^{-12} F/m), V_{TFL} is the trap-filled limit voltage, V_b is the voltage at which the charge transport transitions from a trap-filling regime to an SCLC regime, J_D is the current density at V_b point, μ is the charge carrier mobility, and L is the thickness of the $CsPbBr_3$ NC film (L = 108.2 nm for DDA-Br-capped $CsPbBr_3$ NCs, and L = 116.5 nm for TTP-Br capped $CsPbBr_3$ NCs respectively).

Table S10. Hole carrier mobility of the DDA-Br-capped, and TTP-Br-capped $CsPbBr_3$ NC films from the fits of the SCLC curves.

**$CsPbBr_3$ NCs**	V_{TFL} (V)	V_b (V)	L (nm)	μ ($cm^2V^{-1}s^{-1}$)
**DDA-Br**	0.36	1.21	108.2	6.87×10^{-7}
**TTP-Br**	0.27	1.0	116.5	1.43×10^{-6}

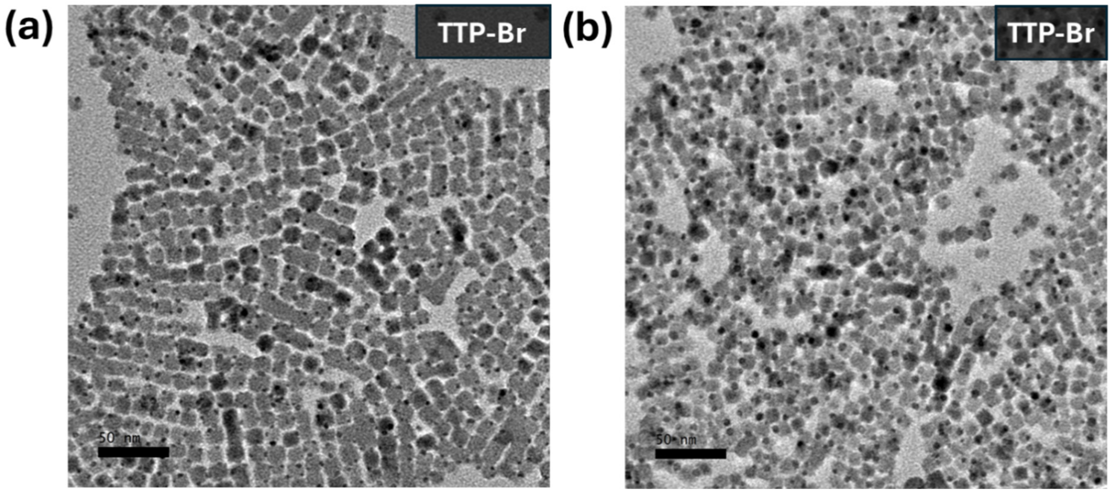

Figure S42. TEM micrographs of TTP-Br-capped $CsPbBr_3$ NCs, (a) after the second treatment (2.5 mM) followed by washing, and (b) after the third treatment (2.5 mM) followed by washing.

References

(1) Imran, M.; Ijaz, P.; Goldoni, L.; Maggioni, D.; Petralanda, U.; Prato, M.; Almeida, G.; Infante, I.; Manna, L. Simultaneous cationic and anionic ligand exchange for colloidally stable $CsPbBr_3$ nanocrystals. *ACS Energy Letters* **2019**, *4* (4), 819-824.

(2) Zaccaria, F.; Zhang, B.; Goldoni, L.; Imran, M.; Zito, J.; van Beek, B.; Lauciello, S.; De Trizio, L.; Manna, L.; Infante, I. The reactivity of $CsPbBr_3$ nanocrystals toward acid/base ligands. *ACS Nano* **2022**, *16* (1), 1444-1455.

(3) Dang, Z.; Shamsi, J.; Palazon, F.; Imran, M.; Akkerman, Q. A.; Park, S.; Bertoni, G.; Prato, M.; Brescia, R.; Manna, L. In situ transmission electron microscopy study of electron beam-induced transformations in colloidal cesium lead halide perovskite nanocrystals. *ACS Nano* **2017**, *11* (2), 2124-2132.

(4) Fairley, N.; Fernandez, V.; Richard-Plouet, M.; Guillot-Deudon, C.; Walton, J.; Smith, E.; Flahaut, D.; Greiner, M.; Biesinger, M.; Tougaard, S.; Morgan, D.; Baltrusaitis, J. Systematic and collaborative approach to problem solving using X-ray photoelectron spectroscopy. *Applied Surface Science Advances* **2021**, *5*, 100112.

(5) D'Andrade, B. W.; Datta, S.; Forrest, S. R.; Djurovich, P.; Polikarpov, E.; Thompson, M. E. Relationship between the ionization and oxidation potentials of molecular organic semiconductors. *Organic Electronics* **2005**, *6* (1), 11-20.

(6) Wu, P. S.; Otting, G. Rapid pulse length determination in high-resolution NMR. *Journal of Magnetic Resonance* **2005**, *176* (1), 115-119.

(7) Wider, G.; Dreier, L. Measuring protein concentrations by NMR spectroscopy. *Journal of the American Chemical Society* **2006**, *128* (8), 2571-2576.

(8) Perdew, J. P.; Burke, K.; Ernzerhof, M. Generalized gradient approximation made simple. *Physical Review Letters* **1996**, *77* (18), 3865.

(9) VandeVondele, J.; Hutter, J. Gaussian basis sets for accurate calculations on molecular systems in gas and condensed phases. *The Journal of Chemical Physics* **2007**, *127* (11).

(10) Hutter, J.; Iannuzzi, M.; Schiffmann, F.; VandeVondele, J. cp2k: atomistic simulations of condensed matter systems. *Wiley Interdisciplinary Reviews: Computational Molecular Science* **2014**, *4* (1), 15-25.

(11) ten Brinck, S.; Infante, I. Surface termination, morphology, and bright photoluminescence of cesium lead halide perovskite nanocrystals. *ACS Energy Letters* **2016**, *1* (6), 1266-1272.

(12) Bodnarchuk, M. I.; Boehme, S. C.; Ten Brinck, S.; Bernasconi, C.; Shynkarenko, Y.; Krieg, F.; Widmer, R.; Aeschlimann, B.; Günther, D.; Kovalenko, M. V.; Infante, I.

Rationalizing and controlling the surface structure and electronic passivation of cesium lead halide nanocrystals. *ACS Energy Letters* **2018**, *4* (1), 63-74.
(13) Rodová, M.; Brožek, J.; Knížek, K.; Nitsch, K. Phase transitions in ternary caesium lead bromide. *Journal of Thermal Analysis and Calorimetry* **2003**, *71*, 667-673.
(14) Kim, J. S.; Heo, J.-M.; Park, G.-S.; Woo, S.-J.; Cho, C.; Yun, H. J.; Kim, D.-H.; Park, J.; Lee, S.-C.; Park, S.-H.; Yoon, E.; Greenham, N. C.; Lee, T. -W. Ultra-bright, efficient and stable perovskite light-emitting diodes. *Nature* **2022**, *611* (7937), 688-694.
(15) Yang, F.; Zeng, Q.; Dong, W.; Kang, C.; Qu, Z.; Zhao, Y.; Wei, H.; Zheng, W.; Zhang, X.; Yang, B. Rational adjustment to interfacial interaction with carbonized polymer dots enabling efficient large-area perovskite light-emitting diodes. *Light: Science & Applications* **2023**, *12* (1), 119.
